# Pharmacologic inhibition of IRE1**α**-dependent decay protects alveolar epithelial identity and prevents pulmonary fibrosis in mice

**DOI:** 10.1172/JCI184522

**Published:** 2025-10-15

**Authors:** Vincent C. Auyeung, Tavienne L. Steinberg, Alina Olivier, Luka Suzuki, Mary E. Moreno, Imran S. Khan, Michael S. Downey, Maike Thamsen, Lu Guo, Dustin J. Maly, Bradley J. Backes, Dean Sheppard, Feroz R. Papa

**Affiliations:** 1 Division of Pulmonary, Critical Care, Allergy, and Sleep Medicine,; 2 Department of Medicine,; 3 Diabetes Center,; 4 Quantitative Biosciences Institute (QBI),; 5 Cardiovascular Research Institute,; 6 Division of Neonatology, Department of Pediatrics, UCSF, San Francisco, California, USA.; 7 Department of Chemistry, University of Washington, Seattle, Washington, USA.

**Keywords:** Pulmonology, Stem cells, Therapeutics, Cell stress, Fibrosis, Protein kinases

## Abstract

Stress-induced epithelial plasticity is central to lung regeneration, fibrosis, and malignancy, but how cellular stress leads to differentiation is incompletely understood. Here, we found a central role for IRE1α, a conserved mediator of the unfolded protein response (UPR), in stimulating the plasticity of alveolar type 2 (AT2) cells. In single-cell RNA-seq, IRE1α activity was associated with loss of AT2 identity and progression toward a damage-associated transitional state unique to fibrosis. AT2 plasticity required destructive regulated IRE1α-dependent decay (RIDD), which we demonstrated by deploying PAIR2, a kinase modulator that inhibits RIDD while preserving IRE1α’s adaptive *XBP1* mRNA splicing activity. In vivo, selective inhibition of RIDD with PAIR2 reduced AT2 differentiation into profibrotic transitional cells and protected mice from bleomycin-induced pulmonary fibrosis. Mechanistically, we identified the *Fgfr2* mRNA as a direct and regulated substrate for IRE1α’s RNase in primary AT2 cells and in a biochemically reconstituted cell-free system. Loss of Fgf signaling caused AT2 differentiation, while gain of signaling protected cells from IRE1α-induced differentiation. We propose that IRE1α downregulates Fgf signaling through RIDD, provoking loss of AT2 identity and differentiation towards a profibrotic phenotype. Thus, IRE1α’s RIDD activity emerges as a novel target for treatment of pulmonary fibrosis and potentially other diseases driven by aberrant epithelial cell plasticity.

## Abstract 2

The study dissects the molecular mechanisms of IRE1<span>α</span> signaling in alveolar type 2 (AT2) cells and pulmonary fibrosis and identifies a therapeutic strategy for kinase modulators to selectively inhibit IRE1α’s pathological mechanism while preserving its homeostatic functions.

## Introduction

Successful repair of epithelial tissues after injury requires progenitor cells to differentiate into new mature epithelial cells. In tissues where they are present, quiescent resident stem cells are mobilized to proliferate and differentiate. In many tissues, previously differentiated epithelial cells can proliferate and dedifferentiate into a transitional state, followed by redifferentiation into new epithelial cells in a phenomenon called plasticity.

In the alveolar epithelium, diverse insults cause alveolar type 2 (AT2) cells to transition into damage-associated transient progenitors (DATPs), which can differentiate into alveolar type 1 (AT1) cells, the flattened epithelial cells that comprise the vast majority of the gas exchange surface area of the lung (1–4). Recently, it has been appreciated that DATP cells accumulate in severe acute lung injury and fibrotic lung disease, leading to the speculation that the failure of cells to exit the transitional state causes them to promote a fibrotic microenvironment (5). Indeed, in experimental lung fibrosis, DATPs express inflammatory chemokines and integrin αvβ6, which, in turn, activates the fibroblast-activating signal TGF-β (6). Thus, DATPs could be key players in the fibrotic niche.

However, the molecular mechanisms that link injury to loss of AT2 identity and then differentiation — or its failure in fibrosis — are unclear. DATPs are characterized by activation of multiple injury/stress response programs, including those associated with DNA damage, hypoxia, unfolded protein, senescence, and inflammation. Activation of these programs is evident in DATPs isolated from diverse lung injury models with different mechanisms of injury, suggesting that lung epithelial cells respond to injury by coordinated engagement of multiple intracellular signaling programs (1, 2, 4).

One of these programs is the unfolded protein response (UPR), an ancient cellular response to protein folding stress in the endoplasmic reticulum (ER stress). IRE1α is an ER transmembrane protein conserved throughout eukaryotes that senses misfolded proteins in the ER lumen and initiates cellular efforts to regain protein folding homeostasis through its cytosolic kinase and RNase domains (7). In addition to their ancient functions in maintaining the folding and maturation of secretory proteins, IRE1α and the UPR are extensively cross wired into other ancient cellular stress programs, including those activated in DATPs, suggesting that the UPR might also regulate AT2 plasticity. Consistent with this cross wiring, we recently showed that IRE1α is activated in DATPs in the bleomycin model of fibrosis, and that IRE1α loss of function dampened TGF-β, DNA damage, and senescence-associated phenotypes in DATPs. Correspondingly, IRE1α loss of function decreased DATP accumulation and decreased fibrotic matrix deposition while enhancing repair (6). In support of this notion, a mutation in a major AT2 secretory cargo protein, surfactant protein C (SPC), triggers ER stress and induces AT2 differentiation into DATPs in an IRE1α-dependent manner (8).

The molecular pathways downstream of IRE1α are complex. Upon activation of IRE1α’s kinase, its RNase induces gene expression changes by facilitating the noncanonical splicing and translation of the messenger RNA encoding the transcription factor Xbp1 (9, 10) or by cleaving micro- and messenger RNAs, leading to their degradation, in a process called regulated IRE1α dependent decay (RIDD) (11, 12). These two activities are catalyzed by distinct conformations and oligomerization states of the kinase/RNase domains, as evidenced by chemical probing, mutational analysis, and structural studies (11, 13, 14).

Here, we deploy ATP-competitive kinase modulator compounds to mechanistically dissect IRE1α’s *Xbp1* splicing and RIDD activities in AT2 differentiation and fibrosis. Using single-cell RNA-seq of injured mouse lungs, we identify a subset of cells we call fibrotic DATPs that emerge in models of fibrosis, but not in normal development or repair. These cells prominently express a signature of IRE1α activation. Using a kinase inhibitor and activator, we find that IRE1α activation is necessary and sufficient for the AT2-to-DATP transition. We use a partial antagonist of the IRE1α kinase — PAIR2 — which inhibits destructive RIDD while preserving adaptive *Xbp1* splicing (15) and find that the AT2-to-DATP transition specifically required RIDD. In mice, PAIR2 is highly effective in preventing bleomycin-induced pulmonary fibrosis. We further identify *Fgfr2* as a bona fide RIDD target, both in primary cells and cell-free systems, thus linking IRE1α pathological activation to Fgf signaling and maintenance of AT2 identity, a regulatory circuit that is conserved in other endodermal lineages. Our findings suggest that pharmacologic inhibition of RIDD may provide a precision approach for treatment of pulmonary fibrosis and other diseases associated with aberrant epithelial plasticity.

## Results

### Fibrotic DATPs emerge after bleomycin injury and are characterized by IRE1α activation.

Recent studies have described a transitional state when AT2 cells differentiate into new AT1 cells (1–4). We and others previously proposed that transitional states accumulate as DATPs in the injured lung as part of a delayed, or failed, program of lung regeneration (5, 16). Substantial accumulations of transitional state cells are found in a range of settings, some associated with fibrosis and some not, including during normal postnatal lung growth, surgical pneumonectomy, and lung injury from COVID19 (17–20). These findings suggest that the transitional state may be heterogeneous: despite superficial similarities in marker gene expression, different cells broadly categorized as “transitional” may enact different cellular programs and may not share the same fate (21, 22).

**Figure 1:**
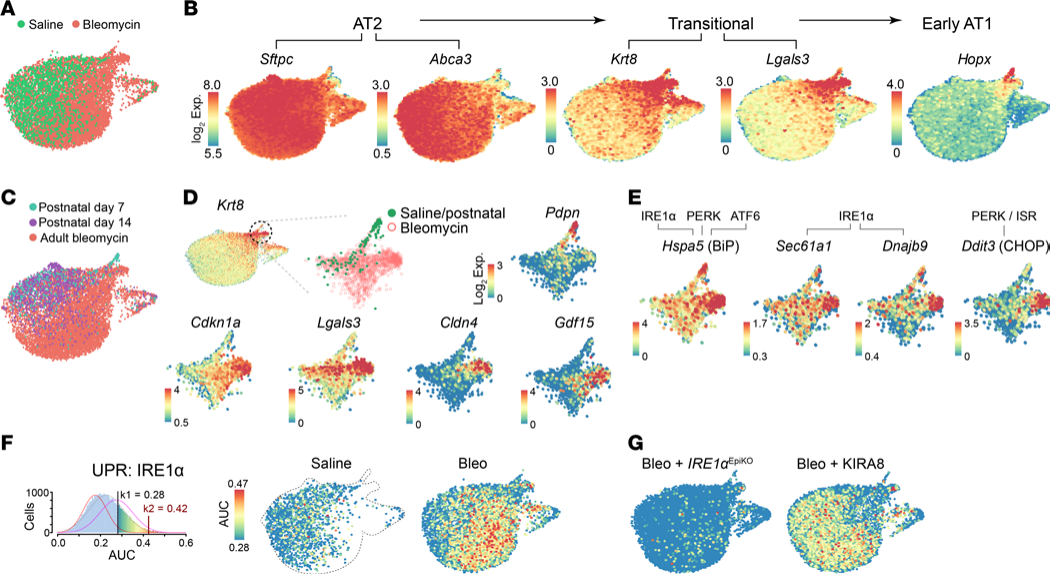
An IRE1α-active subpopulation of fibrotic DATPs emerges after bleomycin injury but not in normal development. (**A**) Single-cell sequencing and uniform manifold approximation and projection (UMAP) of epithelial cells from adult mice exposed to saline (*n* = 2 mice) or bleomycin (*n* = 3 mice). (**B**) Expression of AT2, transitional state, and early AT1 markers. (**C**) Integration of cells from postnatal alveolar growth at postnatal day 7 (*n* = 2 mice) and day 14 (*n* = 2 mice) with adult cells in **A**. (**D**) UMAP detail of cells with high *Krt8* expression with overlay of an AT1 marker (*Pdpn*) and markers of DATPs that emerge after bleomycin injury. (**E**) UMAP of cells as in **D** with overlay of expression of genes associated with the IRE1α and PERK arms of the unfolded protein and integrated stress responses (ISR). (**F**) Inference of IRE1α activity based on area-under-curve (AUC) analysis of genes upregulated by IRE1α, overlaid on a UMAP of saline or bleomycin-exposed cells. Based on the distribution of scores (left), IRE1α is considered inactive in cells with AUC < 0.28. (**G**) IRE1α activity scores in cells from bleomycin exposed mice with IRE1α EpiKO, or treated with an IRE1α kinase inhibitor (KIRA8).

To further understand the distinctions between the normal/regenerative and fibrotic program, we performed single-cell transcriptome profiling of lung epithelial cells in nonfibrotic conditions and after bleomycin injury. To capture potentially subtle differences across these conditions, we sequenced more than 80,000 sorted epithelial cells at a target depth of 30,000 reads/cell. Sequenced cells were filtered for quality and to exclude small numbers of airway and nonepithelial cells (Supplemental Figure 1A; supplemental material available online with this article; https://doi.org/10.1172/JCI184522DS1). In mice exposed to a single saline aspiration, our data revealed a spectrum of transcriptional states between AT2 and AT1 cells, including cells expressing markers of DATPs (*Krt7*, *Krt8*, *Lgals3*, *Sfn*) (Figure 1, A and B, and Supplemental Figure 1, A and B). To further define a reparative transitional state, we performed single cell sequencing of uninjured mouse lungs from postnatal days 7 and 14, during the peak of postnatal alveolar growth (also known as “secondary septation”), due in part to AT2 differentiation into new AT1 cells (17). Cells from postnatal lungs mapped onto the same transitional states (Figure 1C). Cells arrayed along the normal transition had relatively increased expression of core Yap/Taz transcriptional target genes (*Lats2*, *Amotl2*, and *Myof*) (Supplemental Figure 1B) (23), in line with the importance of Hippo signaling in postnatal AT1 specification (24–26). Bleomycin injury triggered the emergence of transcriptional states absent in control and postnatal lungs (Figure 1, A and C). Cells in bleomycin-injured lungs expressed higher levels of markers associated with epithelial injury and inflammation, such as the alarmin *Il33*, the senescence-associated cell-cycle regulator *Cdkn1a* (p21), and the proapoptotic factor *Bax* (Supplemental Figure 1B).

We looked closely at cells expressing *Krt8* and found a unique cluster of cells that emerged after bleomycin injury (Figure 1D). These cells are essentially absent in saline-exposed mice and postnatal alveolar growth (Figure 1D). Compared with cells in the transitional state from saline control and postnatal mice, these cells were marked by high expression of *Cdkn1a* (encoding p21) and uniquely expressed *Cldn4* and *Gdf15*, a biomarker correlated with poor outcomes in human lung fibrosis (27, 28) (Figure 1D).

The unfolded protein response (UPR), a cellular response to protein folding stress, and a related stress response called the integrated stress response (ISR), are both implicated in the pathogenesis of lung fibrosis (29, 30). Cells from bleomycin-exposed mice were prominently marked by canonical genes associated with multiple arms of the UPR and ISR (Figure 1E). In particular, we found high expression of genes regulated by IRE1α, a deeply conserved mediator of the UPR that we and others previously showed to be important in the accumulation of DATPs in fibrosis (6, 8) (Figure 1E).

To more comprehensively evaluate IRE1α activity, we used an expression signature of genes with IRE1α-dependent upregulation after pharmacologic ER stress (31) and mapped AUC expression scores, a measure of relative regulon activity (32). Bleomycin injury was associated with increased IRE1α AUC scores in AT2 cell states that emerged after bleomycin exposure (Figure 1F). We also performed single-cell sequencing on mice with conditional knockout of IRE1α in the lung epithelium (*Shh*^Cre/+^ *Ern1*^flox/flox^, hereafter *IRE1α*^EpiKO^), and WT mice exposed to bleomycin and treated with a pharmacologic kinase inhibitor of IRE1α (KIRA8). This ATP-competitive inhibitor belongs to a class of kinase inhibiting RNase attenuator (KIRA) compounds that block all the downstream effector mechanisms of IRE1α; KIRA8 is highly specific for IRE1α with nanomolar potency (33). Both IRE1α kinase inhibition and genetic loss of function in the epithelium reduced AUC scores for the IRE1α gene signature (Figure 1G), supporting the specificity of the gene signature in the lung.

We applied this regulon analysis and the IRE1α signature to single-cell sequencing of the epithelium from human idiopathic pulmonary fibrosis (IPF) a disease of progressive and fatal lung fibrosis associated with the emergence of abnormal epithelial cell populations (34, 35). We found high AUC expression scores for IRE1α in IPF AT2 cells (Supplemental Figure 2, A and B), along with airway secretory cells. We also found IRE1α activity scores above threshold AUC expression scores in a unique population of “aberrant basaloid” cells (Supplemental Figure 2A) that express *KRT17* without *KRT5* (Supplemental Figure 2B). Cell clusters with high IRE1α activity generally also scored highly for general ER stress and activation of PERK, another sensor of ER unfolded proteins that triggers the ISR (Supplemental Figure 2, A and B).

### IRE1α-active fibrotic DATPs occur in multiple models of lung fibrosis but not in normal development or repair.

**Figure 2:**
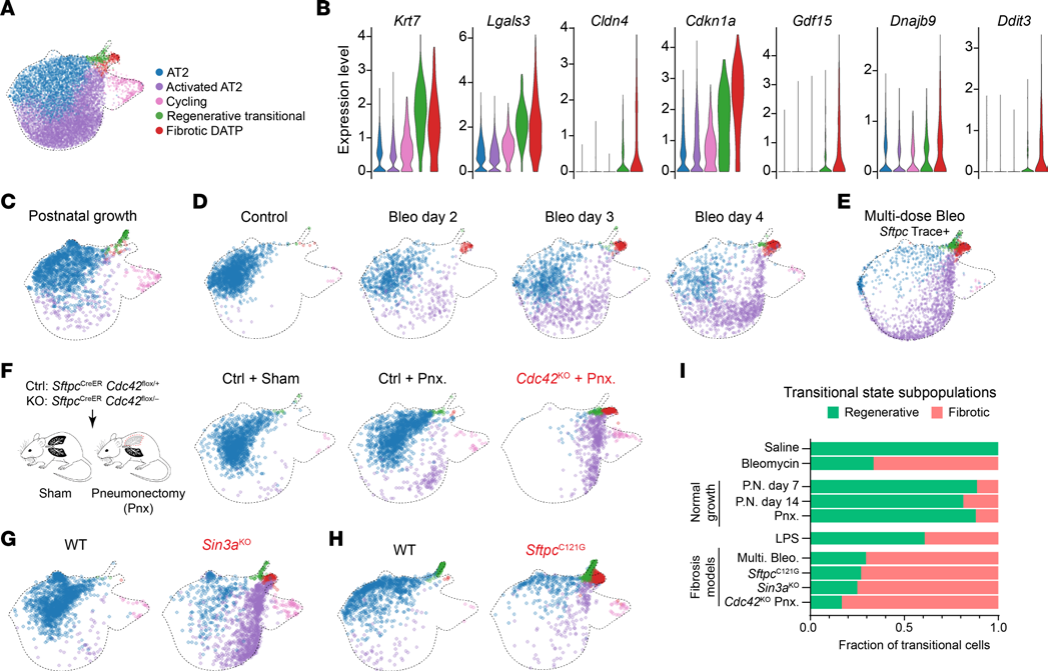
Fibrotic DATPs occur in multiple models of lung fibrosis but not in models of normal repair. (**A**) UMAP of cells from bleomycin-exposed mice with overlay of identified clusters. (**B**) Violin plot of regenerative transitional and fibrotic DATP markers in cells from bleomycin-exposed mice. (**C**) UMAP of cells from postnatal day 7 and day 14 mice with overlay of identified clusters. (**D**) Integrated reference mapping of single cell sequencing from a bleomycin timecourse (https://github.com/theislab/2019_Strunz). (**E**) Integrated reference mapping of *Sftpc* lineage-traced cells from mice in the multiple-dose bleomycin model (GSE243252). (**F**) Integrated reference mapping of single-cell sequencing from pneumonectomy (Pnx) in control or *Cdc42* knockout mice (GSE138585). *Cdc42* knockout mice develop fibrosis only after pneumonectomy. (**G**) Integrated reference mapping of single cell sequencing from mice with fibrosis caused by conditional knockout of *Sin3a* in AT2 cells (GSE132910). (**H**) Integrated reference mapping of single cell sequencing from mice with knock-in of a mutation identified in human fibrosis patients at the Spc locus (*Sftpc*^C121G^) (GSE189479). (**I**) Quantification of the proportion of transitional cells belonging to the regenerative cluster versus the fibrotic DATP cluster in mouse models of growth/repair or fibrosis.

To further characterize the bleomycin-emergent DATPs, we further subclustered the *Krt8*-expressing cells into 2 clusters. Cells from saline control mice and postnatal alveolar growth mapped primarily to a cluster we term “regenerative” (Figure 2, A–C). The other captured the cells that emerge after bleomycin injury that we term the “fibrotic DATP” cluster (Figure 2, A and B). Both clusters share elevated expression of *Krt7* and *Lgals3*. Cells in the “fibrotic DATP” cluster had higher expression of *Cldn4, Cdkn1a, Gdf15,* and the UPR/ISR markers *Dnajb9* and *Ddit3* (Figure 2B). After injury, cells in this cluster emerge by day 2, and become abundant by day 4 after bleomycin exposure (Figure 2D).

We performed differential gene expression testing between cells on the normal/regenerative transition and fibrotic DATPs and inferred regulatory pathways that distinguish them using Ingenuity Pathway Analysis (Supplemental Figure 3A). In the normal transition, we identified upstream regulators with well-established roles in AT1 differentiation, including those involved in mechanosensing and Hippo signaling (p38 MAPK, TEAD transcription factors) and signals from the mesenchyme (WNT3A, Notch, FGF10). Other regulators with strong statistical signals in the normal trajectory have less well-established roles, like the potassium channel KCNJ2 and components of the mTOR pathway (PTEN, TSC1, RICTOR), or, like TGF-β, have complex roles in both normal differentiation and injury. In fibrotic DATPs, we identified regulators associated with DNA damage and senescence (DDX5, RB1, CDKN1A, CDKN2A, ATM), inflammation (IL33, TLR4, IL1, STING1), and epithelial stress (GDF15, HSPA5).

In embryonic lung development and postnatal alveolar growth, cells with transcriptomic similarity to adult transitional states have been described. These cells share some markers with both our regenerative and fibrotic populations and are predicted to have high AT1 differentiation potential (17). We examined single-cell sequencing of sorted Icam1^+^ cells from E14.5, which enriched for cells situated spatially and transcriptionally between *Sox9*^+^ distal epithelium and *Sox2*^+^ proximal epithelium (36). We found only a few cells expressing the fibrotic cluster and UPR markers, with slight enrichment for *Cldn4* and *Cdkn1a* in the *Icam1*^high^ cluster (Supplemental Figure 3B). Conversely, *Icam1* did not distinguish regenerative from fibrotic populations in bleomycin-injured adult lungs, as we found widespread expression in both populations and in activated AT2 cells (Supplemental Figure 3C). Postnatally, we examined our P7 and P14 data carefully and found a few cells that were assigned to the “fibrotic DATP” cluster (Figure 2C). However, these cells had lower expression of these markers than their bleomycin-induced counterparts (Supplemental Figure 3D). These results suggest that the fibrotic DATP state is normally rare and unlikely to be required for lung development or postnatal growth.

We previously showed that the emergence of DATPs in bleomycin-induced fibrosis required epithelial IRE1α (6). In contrast, the normal transition was associated with lower IRE1α-induced gene expression and UPR/ISR pathway scores (Figure 2B and Supplemental Figure 3, A and D). Therefore, we wondered whether IRE1α is required for the normal/regenerative transition. Disruption of normal AT2-to-AT1 differentiation during postnatal alveolar growth leads to reduced gas exchange surface area and alveolar enlargement, indicated morphometrically by increased mean linear intercept on histologic sections. We did not find any differences between *IRE1α*^EpiKO^ and flox-control mice raised in room air conditions (Supplemental Figure 3E). Since subtle deficits in differentiation might be more apparent after perturbation, we also exposed mice to hyperoxia, which impairs alveolar growth without fibrosis, but still did not find any differences between control and *IRE1α*^EpiKO^ mice (Supplemental Figure 3E). Thus, IRE1α activity is neither associated with nor required for cells differentiating along the normal trajectory.

We next wondered whether the “fibrotic DATP” cluster was unique to single-dose bleomycin injury, or more generally associated with fibrosis. We gathered published single-cell transcriptomic data from additional models of injury. Repetitive exposure to bleomycin causes more persistent and severe fibrosis in mice compared with a single-dose injury. *Sftpc* lineage-traced cells from this model (37) mapped to the fibrotic DATP cluster, confirming that AT2 cells contribute to the fibrotic DATP population in more severe fibrosis (Figure 2E and Supplemental Figure 4, A and B).

Pneumonectomy is associated with effective alveolar growth without fibrosis (38) and was associated with the emergence of a cluster of cells expressing *Krt7* and *Lgals3*, but not *Cldn4* (Supplemental Figure 4A), which were most similar to our regenerative transitional cluster based on marker gene similarity (Supplemental Figure 4B) and UMAP projection (Figure 2F), at least at the relatively late timepoint in that dataset. In contrast, mice with AT2-specific *Cdc42* knockout are defective in mechanosignaling, and pneumonectomy causes nonresolving, progressive fibrosis, resulting in death over several months (18). This single genetic defect provoked the emergence of a *Cldn4*^+^ fibrotic DATP cluster (Figure 2F and Supplemental Figure 4, A and B).

AT2-specific knockout of the chromatin maintenance factor *Sin3a* (*Sin3a*^KO^) causes widespread chromatin dysregulation and DNA damage signaling, followed by nonresolving, progressive fibrosis resulting in death over several weeks (39). Cells in this model mapped onto the fibrotic DATP cluster (Figure 2G and Supplemental 4, A and B).

To establish that UPR signaling is cell-autonomously sufficient to produce fibrotic DATPs, we analyzed cells from mice with a cysteine mutation in *Sftpc* (*Sftpc^C121G^*), which is found in patients with familial fibrosis and causes UPR activation in AT2 cells and lung fibrosis (8). Cells from this model likewise mapped to the fibrotic DATP cluster, indicating that AT2-autonomous ER stress can trigger differentiation into fibrotic DATPs (Figure 2H and Supplemental Figure 4, A and B).

For each of these models, we quantified the proportion of cells mapping to the normal transitional state or to the fibrotic DATP cluster. In postnatal alveolar growth and postpneumonectomy repair, the vast majority of cells mapped to the normal transition. Conversely, the majority of cells from fibrosis models mapped to the fibrotic DATP cluster (Figure 2I).

Fibrotic DATP cells were not restricted to models predominantly characterized by fibrosis. We also analyzed *Sftpc* lineage-traced cells from a model of acute lung injury stimulated by intratracheal instillation of lipopolysaccharide (LPS) (40). Cells from this model largely mapped to the normal transition, consistent with alveolar regeneration and AT2-to-AT1 differentiation in this model (Figure 2I and Supplemental Figure 4C). Nonetheless, a significant minority mapped to the fibrotic DATP cluster (Figure 2I). These cells shared markers with murine “highly senescent basaloid” cells that localize to focal regions of fibrosis in the LPS model and may have limited capacity to differentiate into AT1 cells (21). Fibrotic DATPs were also similar to “*Cldn4*^high^” cells in organoid culture of *Nkx2-1* knockout alveolar epithelial progenitors (Supplemental Figure 4D), which express markers of cellular stress — including activation of the UPR/ISR — and accumulate at the expense of normal alveolar identities including AT2 and AT1 (41). Together, these results suggest that fibrotic DATPs are a distinct subpopulation, associated with fibrotic microenvironments in multiple models, with questionable AT1 differentiation potential.

### AT2-to-DATP transition can be decoupled from XBP1 splicing and instead requires RIDD.

**Figure 3:**
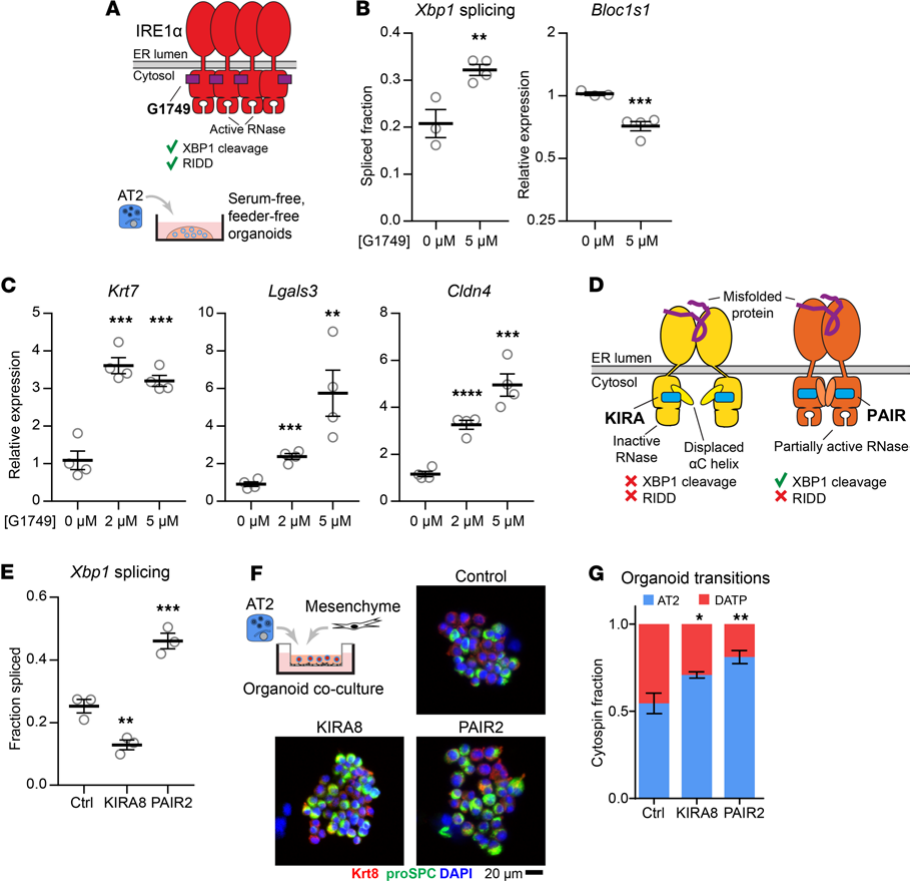
AT2 to DATP transition requires RIDD. (**A**) Schematic of G1749 binding to the IRE1α kinase domain and the stimulated effector activities. (**B**) IRE1α activation by G1749 in serum-free, feeder-free AT2 organoids based on increased Xbp1 splicing and decreased expression of *Bloc1s1*, a canonical RIDD substrate. *Bloc1s1* data plotted on a logarithmic scale. (**C**) Expression of DATP markers *Krt7*, *Lgals3*, and *Cldn4* in serum-free, feeder-free AT2 organoids transiently stimulated with G1749. (**D**) Schematic of KIRA and PAIR binding to the IRE1α kinase domain and the stimulated effector activities. (**E**) *Xbp1* splicing fraction in AT2 organoids cocultured with CD45^–^ CD31^–^ EPCAM^–^ mesenchyme treated with KIRA8 or PAIR2. (**F**) Representative cytospin fields for organoids cocultured and treated as in **E**. (**G**) Quantification of cell transitions in organoids by cytospin and staining for proSPC and Krt8 (*n* = 3 culture wells in each group). Individual culture well replicates shown with mean ± SEM. * *P* < 0.05, ** *P* < 0.01, *** *P* < 0.001 by 1-sided Student’s *t* test.

We noted that a signature of IRE1α activity was prominently upregulated in bleomycin-injured AT2 cells (Figure 1F), and in AT2 cells from human patients with lung fibrosis (Supplemental Figure 2). Since AT2 cells are precursors to mouse DATPs (1–4) and human “aberrant basaloid” cells (42), we next asked whether IRE1α activation in AT2 cells would be sufficient to induce a cell state resembling DATPs. IRE1α can be selectively activated using G1749, an ATP-competitive compound that binds the ATP pocket of the kinase domain and allosterically activates the RNase domain (43) (Figure 3A). G1749-bound IRE1α lacks phosphotransfer activity, as evidenced by reduced autophosphorylation in a cell line overexpressing IRE1α (Supplemental Figure 5A). Therefore, the effects of G1749 activation are mediated through IRE1α’s RNase activity.

We generated AT2 organoids under serum-free, feeder-free conditions optimized for the maintenance of the AT2 identity (44). As expected, exposure of AT2 organoids to G1749 induced both effector activities of the IRE1α RNase: cleavage and splicing of *Xbp1*, and regulated IRE1α-dependent decay (RIDD) of the canonical substrate *Bloc1s1* (also known as *Blos1*) (Figure 3B). G1749 did not induce *Atf4*, the most direct downstream transcriptional target of the PERK arm of the UPR and the integrated stress response, and only weakly induced *Ddit3* (encoding the protein CHOP) (Supplemental Figure 5B), consistent with observations that *Ddit3* induction is partially IRE1α dependent in some contexts (11, 45).

Cells grown in these conditions are resistant to DATP or AT1 transition and can be passaged for several rounds without significant differentiation (44). We asked whether differentiation could be provoked by transient activation of IRE1α. Organoids were exposed to G1749 for 24 hours followed by 3 days of recovery in normal media. G1749 dose-dependently induced the canonical markers of the DATP state: *Krt7*, *Lgals3*, and *Cldn4* (Figure 3C). Upregulation of *Itgb6* (Supplemental Figure 5C) suggested that the cell state induced by G1749 might be able to induce TGF-β release similar to DATPs in vivo, which are the cells principally expressing integrin αvβ6 after fibrotic injury (6). Differentiation was incomplete, since cells did not lose proSPC staining on cytospin (Supplemental Figure 5D). Organoids also minimally expressed *Hopx*, a marker of AT1 cells (Supplemental Figure 5E).

We wondered how IRE1α mechanistically regulates the AT2-to-DATP transition. Once its kinase domain is activated, IRE1α’s RNase adopts an active conformation and cleaves the *Xbp1* mRNA. This triggers an evolutionarily conserved sequence of molecular events: religation of the cleaved *Xbp1* RNA fragments, shifting of the translational reading frame bypassing a premature termination codon, and synthesis of a functional transcription factor that supports protein folding homeostasis (7). Other mRNA substrates cleaved by IRE1α lack the capacity to be religated and are instead degraded in a process called RIDD (11, 12). We previously showed that DATPs have the transcriptional signature of RIDD, but not *Xbp1* splicing, suggesting that RIDD is the critical effector mechanism (6). However, our functional studies could not distinguish *Xbp1* splicing and RIDD, since our previous pharmacologic and genetic approaches inhibited both effector mechanisms.

To segregate *Xbp1* splicing from RIDD, we recently developed ATP-competitive kinase modulators called “partial antagonist of IRE1α’s RNase” (PAIR) compounds (15). Like KIRA8, the compound PAIR2 is highly specific for the IRE1α kinase with nanomolar potency. However, PAIR2 only inhibits RIDD while preserving and even enhancing XBP1 splicing (15) (Figure 3D), which we confirmed in AT2 organoids (Figure 3E and Supplemental Figure 5F). These compounds cannot be used together with G1749 because they compete for the same ATP-binding pocket in the IRE1α kinase domain. Instead, we modeled the AT2-to-DATP transition using AT2-mesenchyme coculture organoids (4). Under these culture conditions, AT2 cells lose *Sftpc* expression as they increase expression of DATP markers including *Krt8* (Supplemental Figure 5G). On cytospin staining, about 50% of cells are Krt8^+^ proSPC^low^ DATPs (Figure 3F and Supplemental Figure 5H). Blocking both effector functions of IRE1α with KIRA8 decreased the ratio of Krt8:Sftpc staining (Supplemental Figure 5I) and correspondingly decreased the proportion of cells that have transitioned into Krt8^+^ proSPC^low^ DATPs by cytospin quantification (Figure 3, F and G). These results were similar with PAIR2 treatment, despite enhancement of *Xbp1* splicing (Figure 3, E–G, and Supplemental Figure 5, H and I). Thus, the DATP transition does not appear to be mediated by *Xbp1* splicing, and we infer that this transition is mediated by RIDD. Coupled with the necessity (6) and sufficiency (Figure 3C) of IRE1α activity in the epithelium for differentiation, these results strongly suggest that RIDD operates cell-autonomously in epithelial cells.

### Selective inhibition of RIDD with PAIR2 protects mice from lung fibrosis.

**Figure 4:**
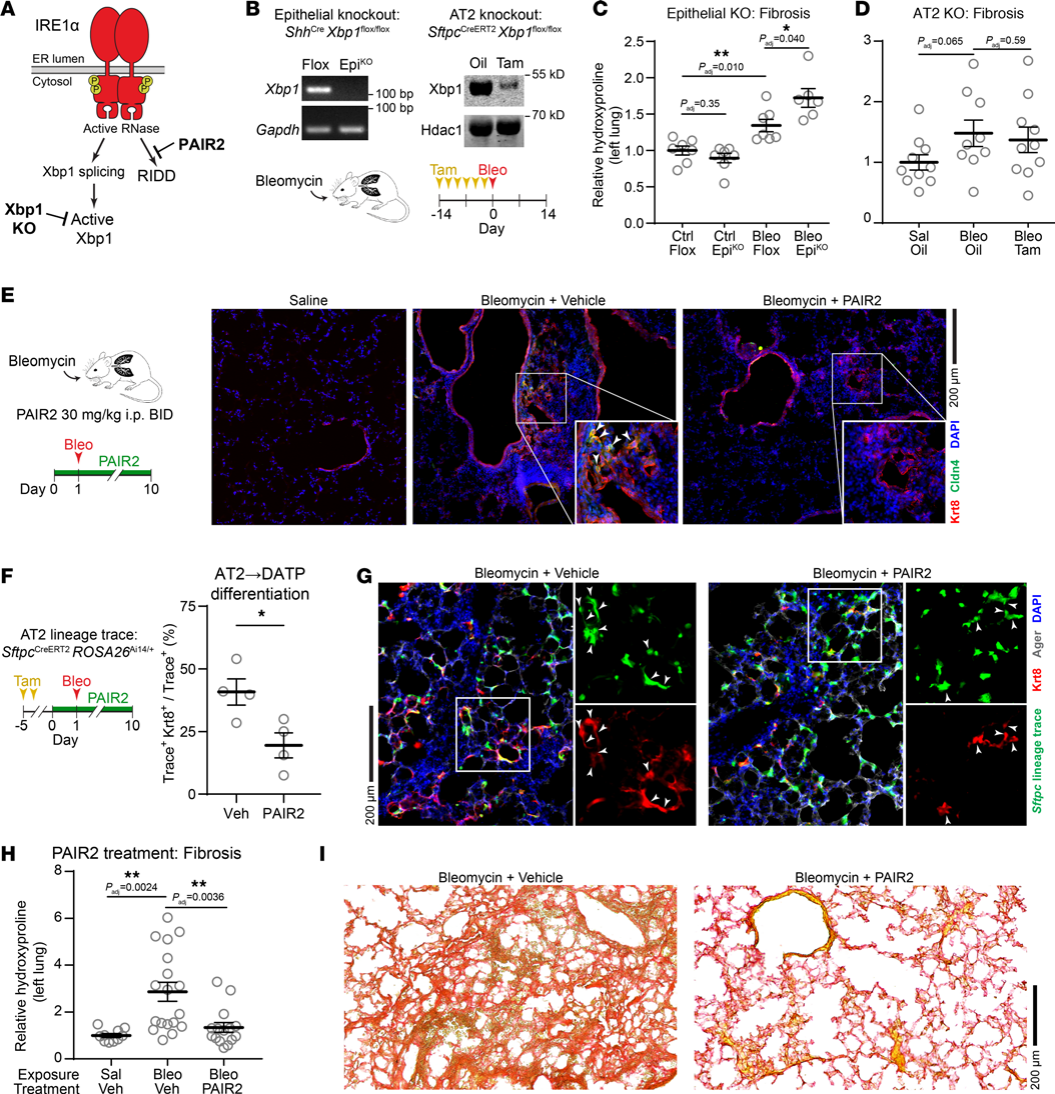
Inhibition of RIDD with PAIR2 protects mice from fibrosis. (**A**) Schematic of IRE1α effector activities and strategies to inhibit each activity in vivo. (**B**) Schematic of *Xbp1* epithelial and AT2 conditional knockout strategies and detection of total *Xbp1* transcript and protein by reverse-transcription PCR and Western blot, respectively. Quantification of fibrosis by hydroxyproline content in lung epithelial knockout mice (**C**) and AT2 knockout mice (**D**) exposed to bleomycin. (**E**) Immunofluorescence staining for Krt8 (red) and Cldn4 (green) at day 10 after bleomycin exposure and treatment with PAIR2 at 30 mg/kg BID. (**F** and **G**) Quantification and staining of Sftpc lineage-traced AT2 cells that have differentiated into trace^+^ Krt8^+^ cells (white arrowheads) at day 10 after bleomycin exposure and treatment with PAIR2 at 30 mg/kg BID. (**H** and **I**) Quantification of fibrosis by hydroxyproline content and picrosirius red staining for fibrillar collagen in mice exposed to bleomycin and treated with PAIR2 at day 14. Individual mouse replicates shown with mean ±SEM. * *P* < 0.05, ** *P* < 0.01 by 1-sided Student’s *t* test with adjusted *P* values where indicated to correct for multiple hypotheses using Šidák’s method. (**E, G,** and **I**) Scale bars: 200 µm.

We previously showed that systemic administration of KIRA8 or conditional knockout of IRE1α protected mice from bleomycin-induced lung fibrosis (6, 46). To interrogate which of IRE1α’s effector functions mediates fibrosis in vivo, we blocked each function genetically or pharmacologically in mice (Figure 4A). Our results with AT2 organoids suggested that *Xbp1* splicing is dispensable for the AT2-to-DATP transition, along with its profibrotic effects in injured regions. We tested this hypothesis by generating conditional knockouts of *Xbp1* in lung epithelial cells using *Shh*^Cre^ (47) and in AT2 cells using *Sftpc^CreERT2^* (48) (Figure 4B). Epithelial cells from knockout mice had grossly normal lungs at baseline (Supplemental Figure 6A) and, as expected, had evidence of increased IRE1α RNase activity (Supplemental Figure 6B) due to loss of *Xbp1*-mediated adaptive signaling (49). In contrast to IRE1α loss of function (6), *Xbp1* knockout failed to protect mice from bleomycin-induced fibrosis, as measured by quantitative hydroxyproline content (Figure 4, C and D). *Xbp1*^EpiKO^ mice had slightly worsened fibrosis (Figure 4C), perhaps due to increased profibrotic signaling through IRE1α (evidenced by increased RNase activity on residual *Xbp1* transcript, [Supplemental Figure 6B]) or other arms of the UPR/ISR (6, 30, 50).

Conversely, since RIDD mediates the DATP transition, we reasoned that pharmacologically blocking IRE1α’s RIDD function might protect mice from fibrosis. We developed a formulation of PAIR2 in a mixture of polyanionic β cyclodextrins and water. While PAIR2 was rapidly cleared from the plasma after intraperitoneal administration, we found steadier tissue levels in the lung after 8 hours, above the concentrations that were efficacious in our organoid cultures (~ 1,200 ng/mL) (Supplemental Figure 6C). As expected, PAIR2 administration did not significantly affect *Xbp1* splicing in the lung, yet derepressed expression of the canonical RIDD target *Bloc1s1*, indicating on-target efficacy of PAIR2 in the lung in vivo (Supplemental Figure 6D).

We exposed mice to bleomycin and twice-daily injections of PAIR2. Since fibrotic DATPs emerge rapidly after bleomycin exposure (Figure 2D), PAIR2 treatment was started on the day before bleomycin exposure. Ten days after bleomycin exposure, at the peak of DATP cell number (1), lungs of PAIR2-treated mice had qualitatively fewer Krt8^+^ cells in the parenchyma, including Krt8^+^ Cldn4^+^ double-positive cells (Figure 4E). Consistent with our results in organoid culture (Figure 3, F and G), *Sftpc*-lineage traced AT2 cells in PAIR2 treated mice were less likely to differentiate into Krt8^+^ transitional cells at day 10 (Figure 4, F and G). These changes corresponded to physiological benefits. PAIR2-treated mice tended toward higher average weight (Supplemental Figure 6E). Most importantly, PAIR2 treatment reduced fibrosis histologically and quantitatively, as measured by picrosirius red staining and hydroxyproline content, respectively, at day 14 (Figure 4, H and I), phenocopying our previous results with complete IRE1α inhibition using KIRA8 or genetic knockout (6, 46). Thus, IRE1α’s RIDD activity accounts for its pathological effects in lung fibrosis.

### RIDD downregulates Fgfr2 and additional genes important to maintenance of AT2 identity.

**Figure 5:**
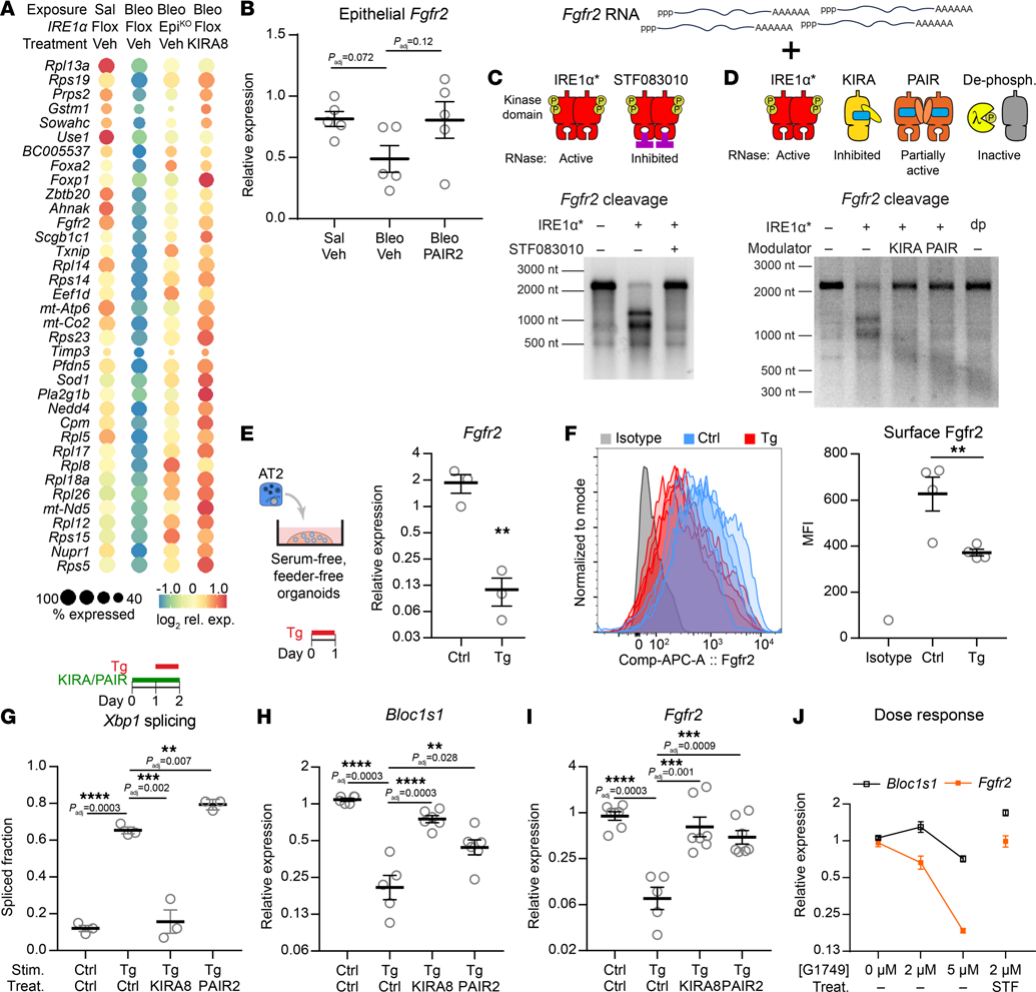
*Fgfr2* is a bona fide substrate for RIDD. (**A**) Dot plot of RIDD candidate genes in cells in the AT2 cluster. Genes were filtered for those that are downregulated after bleomycin injury and whose expression is rescued by IRE1α genetic or pharmacologic loss-of-function. (**B**) Whole-lung expression of the epithelial *Fgfr2* splice isoform (Fgfr2b) after bleomycin exposure and treatment with PAIR2. (**C**) In vitro cleavage of a T7-transcribed *Fgfr2* RNA substrate by IRE1α* with or without the selective IRE1α RNase inhibitor STF083010. (**D**) In vitro cleavage of *Fgfr2* as in **C** after modulating the kinase domain using the complete inhibitor KIRA8, RIDD-selective inhibitor PAIR2, and complete dephosphorylation (dp) by lambda phage phosphatase. (**E**) *Fgfr2* expression in serum-free, feeder free AT2 organoids after stimulation with the ER stress agent thapsigargin (Tg). Values plotted on a logarithmic scale. (**F**) Surface Fgfr2 expression by flow cytometry in AT2 organoids, with quantification of mean fluorescence intensity (MFI). *Xbp1* splicing (**G**), *Bloc1s1* (**H**) and *Fgfr2* (**I**) expression in serum-free, feeder free AT2 organoids after stimulation with the ER stress agent thapsigargin (Tg) and treatment with KIRA8 or PAIR2. Values plotted on a logarithmic scale for **H** and **I**. (**J**) Bloc1s1 and Fgfr2 expression in response to dose-titration of G1749 and reversal with the selective IRE1α RNase inhibitor STF083010. Mean ±SEM shown for visual clarity with *n* = 7 for 0 μM and *n* = 4 for all other doses, plotted on a logarithmic scale. For all other panels, individual mouse or culture well replicates shown with mean ±SEM. * *P* < 0.05, ** *P* < 0.01, *** *P* < 0.001, **** *P* < 0.0001 by 1-sided Student’s *t* test with adjusted *P* values where indicated to correct for multiple hypotheses using Šidák’s method.

Since IRE1α’s RIDD activity promotes the DATP transition and lung fibrosis, we next explored the molecular mechanisms that would connect RIDD to epithelial plasticity. Messenger RNAs regulated by RIDD are cleaved by IRE1α’s RNase domain and subsequently degraded. To identify candidate RIDD substrates in injured AT2 cells, we examined AT2 cells in our single cell sequencing to identify transcripts whose downregulation by injury depended on IRE1α. We stringently required candidate gene expression to be rescued by both the kinase inhibitor KIRA8 and IRE1α^EpiKO^, thereby avoiding candidate genes whose expression might be rescued as a secondary consequence of IRE1α inhibition in other cell types or an unexpected effect of *Shh* haploinsufficiency or Cre recombinase activity. This approach identified 36 candidate genes (Figure 5A). We confirmed that injury downregulated these genes in independent bulk profiling of sorted AT2 cells after bleomycin injury (30) (Supplemental Figure 7A).

The RIDD candidate genes included several with potential consequences for lung epithelial plasticity and fibrosis. *Foxa2* encodes a forkhead family transcription factor that acts as a “pioneer” transcription factor during specification of the endoderm and subsequently suppresses goblet cell fate (51). Another RIDD candidate, *Fgfr2*, encodes a receptor tyrosine kinase that mediates signals from multiple ligands to promote AT2 specification, maintenance, and self-renewal; its loss after injury increases AT2 differentiation into AT1 cells (52–55). Additional candidates with potential relevance to fibrosis included the antioxidant gene *Sod1*, which promotes recovery from oxidative stress by scavenging reactive oxygen species, and *Ahnak*, a regulator of TGF-β signaling.

Given *Fgfr2*’s importance in AT2 plasticity, and translation of its mRNA at the ER membrane, we investigated further whether *Fgfr2* is a bona fide RIDD substrate. The epithelial-specific splice isoform of *Fgfr2* was downregulated by bleomycin injury (Figure 5B), which we confirmed in published bulk transcriptome profiling of sorted AT2 cells after bleomycin injury (30) (Supplemental Figure 7A), and expression tended to be higher in PAIR2-treated mice.

We next generated an AT2-specific *Fgfr2* RNA substrate by reverse transcription of the coding sequence (CDS) from AT2 mRNA followed by T7 RNA polymerase synthesis. Incubation of this substrate with purified recombinant IRE1α kinase/RNase catalytic domains (IRE1α*) revealed a single cleavage site in the CDS (Figure 5, C and D), which could be blocked by STF083010, a selective inhibitor of the IRE1α RNase (56) (Figure 5C). In vitro cleavage depended on allosteric control by IRE1α’s kinase domain, and could be blocked by KIRA8 or dephosphorylation of IRE1α* with lambda phosphatase (Figure 5D). Importantly, in vitro cleavage was blocked by PAIR2, which selectively blocks RIDD but not XBP1 cleavage (15). Thus, *Fgfr2* is a direct and regulated cleavage substrate of IRE1α.

IRE1α-dependent regulation of *Fgfr2* expression in the mouse lung (Figure 5, A and B) is consistent with RIDD, but could be the result of multicellular interactions in the complex fibrotic niche. To establish that IRE1α cell-autonomously downregulates *Fgfr2* in AT2 cells, we turned again to AT2 cells grown in serum-free, feeder-free organoids. The compound thapsigargin induces ER stress and multipathway UPR signaling, evidenced by induction of *Xbp1* splicing downstream of IRE1α (Supplemental Figure 7B) and strong upregulation of *Atf4* and *Ddit3* (encoding CHOP) downstream of PERK (Supplemental Figure 7C). Thapsigargin decreased expression of *Fgfr2* mRNA (Figure 5E), corresponding to reduced cell-surface expression of Fgfr2 by flow cytometry (Figure 5F and Supplemental Figure 7D). Complete inhibition of IRE1α using KIRA8 suppressed *Xbp1* splicing, while selective inhibition of RIDD using PAIR2 did not (Figure 5G). Expression of bona fide RIDD substrates would be expected to be independent of *Xbp1* splicing, as is the case for *Bloc1s1* (Figure 5H). Likewise, *Fgfr2* expression was rescued by both KIRA8 and PAIR2 (Figure 5I), indicating that its downregulation depends on IRE1α kinase conformation, but not on *Xbp1* splicing activity.

We next asked whether *Fgfr2* downregulation in cells depends on IRE1α’s RNase activity. The selective kinase activator compound G1749 is ATP competitive and thus inhibits the kinase domain’s phosphotransfer activity, while simultaneously inducing an active conformation that, in turn, allosterically activates the RNase domain (43). G1749 dose-dependently reduced *Fgfr2* expression, demonstrating that an active kinase conformation is sufficient and does not require phosphotransfer activity (Figure 5J and Supplemental Figure 5A). At both G1749 doses tested, the magnitude of *Fgfr2* downregulation was greater than that of *Bloc1s1*, the canonical RIDD substrate. Inhibiting IRE1α’s RNase using the specific endoribonuclease inhibitor STF083010 rescued *Fgfr2* expression, indicating the necessity of RNase catalytic activity for regulation of *Fgfr2* (Figure 5J). We therefore conclude that *Fgfr2* is a bona fide RIDD target in AT2 cells.

### IRE1α regulates AT2 plasticity through Fgfr2.

**Figure 6:**
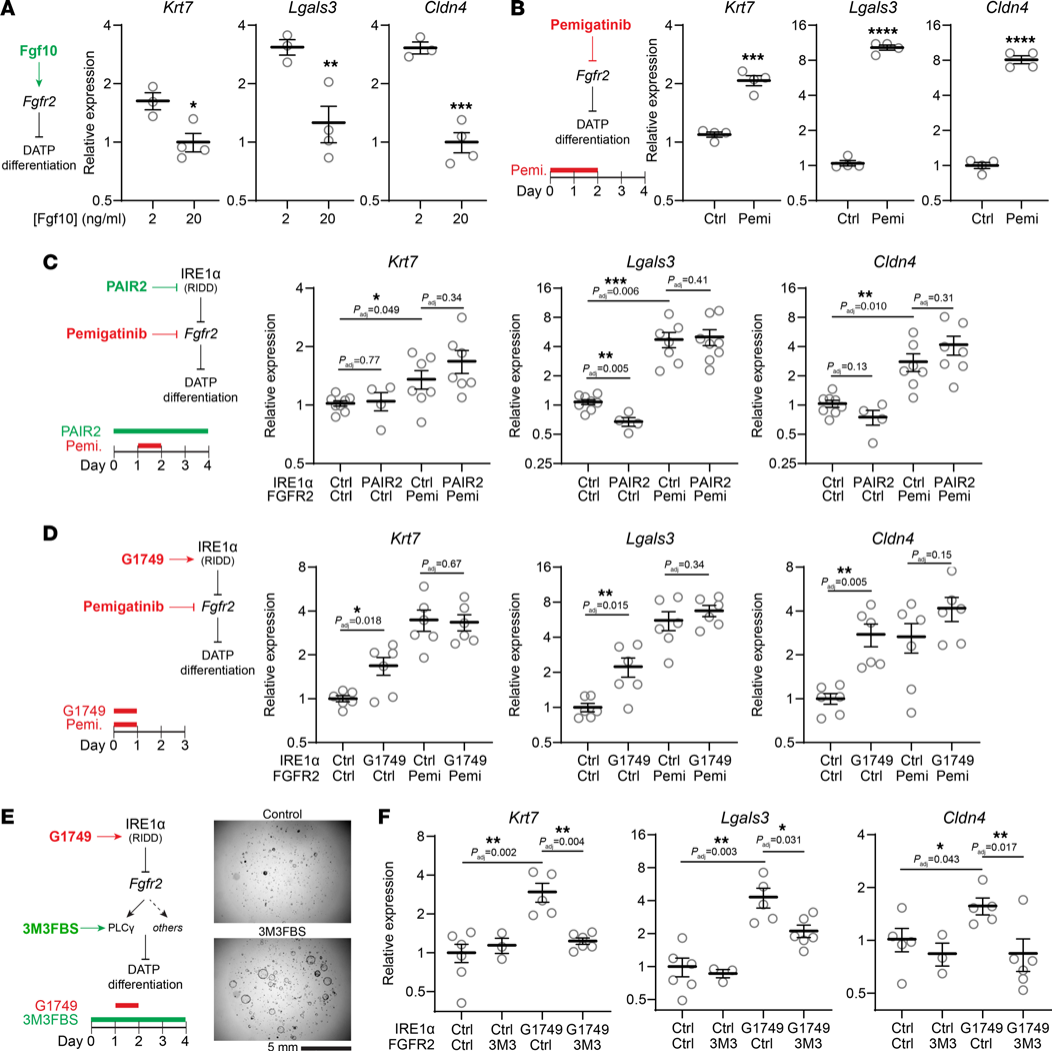
Regulation of *Fgfr2* is necessary and sufficient for IRE1α-induced AT2-to-DATP differentiation. (**A**) Expression of DATP markers *Krt7*, *Lgals3*, and *Cldn4* in serum-free, feeder-free AT2 organoids grown at various concentrations of Fgf10. (**B**) Expression of DATP markers in AT2 organoids after transient pemigatinib exposure for 24 hours followed by 3 days of recovery. (**C** and **D**) Expression of DATP markers in AT2 organoids stimulated with pemigatinib and treated with PAIR2, or double-stimulated with pemigatinib and G1749. (**E**) Schematic of IRE1α activation and rescue by enhanced signaling downstream of *Fgfr2*, and brightfield images of Matrigel droplets containing organoids grown with or without the phospholipase C activator m-3M3FBS. (**F**) Expression of DATP markers in organoids with dual stimulation of IRE1α and phospholipase C. Individual culture well replicates shown with mean ±SEM. All values plotted on a logarithmic scale. * *P* < 0.05, ** *P* < 0.01 by 1-sided Student’s *t* test with adjusted *P* values where indicated to correct for multiple hypotheses using Sidak’s method.

We next asked whether downregulation of *Fgfr2* and the subsequent decrease in Fgf signaling would promote AT2 differentiation. *Fgfr2* is the principal receptor expressed in AT2 cells, and the other isoforms (*Fgfr1*, *Fgfr3*, and *Fgfr4*) are dispensable at rest and after injury (53). In serum-free, feeder-free AT2 organoids, DATP marker expression depended on the concentration of Fgf10, the sole Fgfr2 ligand in this defined culture system (Figure 6A). To abrogate Fgf signaling independently of IRE1α, we used pemigatinib, a tyrosine kinase inhibitor recently approved by the US Food and Drug Administration that is highly specific for Fgf receptors with subnanomolar potency (57). Transient loss of Fgf signaling with a pemigatinib pulse followed by 2 days of recovery induced the canonical markers of the DATP state (Figure 6B), phenocopying the effect of transient IRE1α activation (Figure 3C). In the face of Fgfr blockade, PAIR2 failed to protect AT2 cells from differentiation (Figure 6C). Conversely, IRE1α activation with G1749 did not further enhance differentiation triggered by loss of Fgf signaling (Figure 6D).

We reasoned that enhancement of signaling downstream of Fgfr2 might protect AT2 cells from IRE1α and RIDD-induced differentiation, even if surface Fgfr2 is downregulated. Ligand-bound FGF receptors activate a complex web of pathways including the PI3 kinase, MAP kinase, phospholipase C, and STAT cascades. We found that the phospholipase C activator *m-*3M3FBS (58) increased the growth of organoids grown in serum-free, feeder-free conditions, similar to the effect of increasing Fgf10 concentration (Figure 6E). 3M3FBS did not affect expression of DATP markers at baseline (Figure 6F). As before, IRE1α activation with G1749 induced DATP marker expression, which was rescued by 3M3FBS (Figure 6F). Together, these experiments suggest that, in defined serum-free, feeder-free culture, downregulation of *Fgfr2* is necessary and sufficient for IRE1α’s effect on AT2 differentiation.

IRE1α’s RIDD function is conserved in fission yeast (59), suggesting that its ancient origins predate multicellular animals, and RIDD targets like *Fgfr2* are members of protein families conserved at least across bilaterian animals. We reasoned that the regulatory relationship between IRE1α and RIDD targets like *Fgfr2* would also be ancient, and that the regulatory circuit might be conserved in other epithelia. The conducting airway and alveoli of the lung develop from distinct epithelial lineages (60), and loss of even one copy of *Fgfr2* induces basal cells to transdifferentiate into *Krt8*-expressing “luminal” cells with features of senescence (61). We grew tracheal epithelial cells in serum free, feeder free organoids (Supplemental Figure 8A) and found that, as in AT2 cells, selective IRE1α activation reduced *Fgfr2* expression (Supplemental Figure 8B). Likewise, *Fgfr2* was downregulated by ER stress induced by thapsigargin, which could be rescued with PAIR2 (Supplemental Figure 8C). In another endoderm-derived tissue, the pancreatic islet, signaling through *Fgfr1* and *Fgfr2* have intricate roles in development and maintenance of β islet identity (62–67), and IRE1α hyperactivation and RIDD can contribute to islet cell death and diabetes (11, 33, 68, 69). In isolated human primary islets, selective IRE1α activation reduced *Fgfr2* expression (Supplemental Figure 8D). Thus, the IRE1α-*Fgfr2* circuit may regulate epithelial identity and differentiation in multiple lineages.

## Discussion

**Figure 7:**
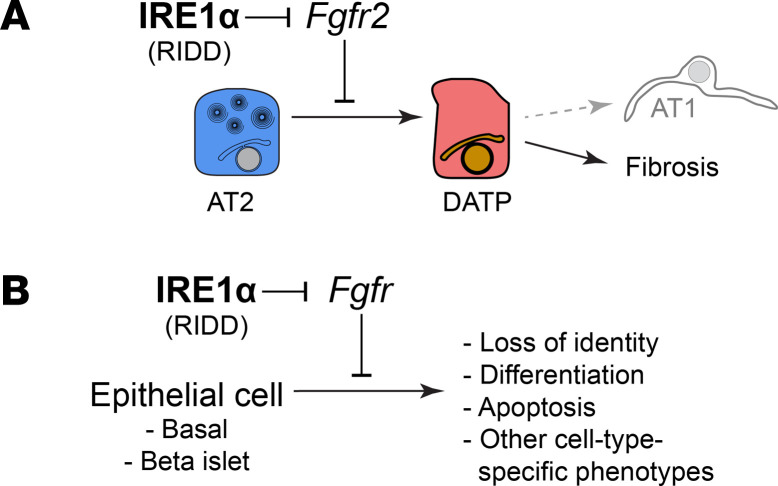
IRE1α promotes AT2 plasticity through downregulation of Fgfr2. (**A**) Model of IRE1α control of AT2 plasticity and promotion of fibrosis by RIDD and downregulation of *Fgfr2*. (**B**) Model of IRE1α modulation of Fgf signaling and potential effects in other epithelial tissues.

Our studies of AT2 plasticity revealed a distinct population of fibrotic DATPs that emerge in multiple lung injury models associated with fibrosis, which exhibit prominent IRE1α activation. We found that IRE1α controls the emergence of DATPs through its RIDD function. Selective blockade of RIDD with a partial IRE1α kinase antagonist, PAIR2, protects mice from bleomycin-induced pulmonary fibrosis. Mechanistically, we demonstrated that *Fgfr2* is a bona fide substrate of IRE1α’s RNase, and in turn, its downregulation contributes to loss of AT2 identity and conversion to the DATP state. We propose a model whereby pathological activation of IRE1α downregulates Fgf signaling, leading to AT2 differentiation into DATPs, which have been shown to promote TGF-β activation and other dysregulated signaling to nucleate a fibrotic niche (1, 2, 6) (Figure 7).

In primary AT2 cells grown in organoids, we found that RIDD-mediated downregulation of *Fgfr2* is both necessary and sufficient for differentiation. An important caveat is that these reductionist culture conditions may not fully recapitulate all of the overlapping cell-intrinsic and microenvironmental cues that maintain AT2 identity in vivo and specify differentiation fate. In the more complex environment of the lung, other RIDD candidate substrates may also contribute to plasticity and fibrosis (Figure 5A), in addition to previously described RIDD targets including Id transcription factors and microRNAs (70–72). Indeed, loss of Fgf signaling could, in principle, result in both the regenerative or fibrotic DATP state (54), suggesting that additional cell-intrinsic or extrinsic cues help select the fibrotic DATP outcome. IRE1α downregulation of other RIDD targets might bias differentiation outcome in favor of the fibrotic DATP state. For example, the RIDD candidate *Foxa2* (Figure 5A) was identified as a regulator of the “unstressed” trajectory leading to AT1 fate, in opposition to the UPR/ISR-activated “stressed” trajectory that resembles our fibrotic DATP state (41). RIDD may also contribute to fibrosis pathology through other cell types, as has been proposed in liver fibrosis (71).

We found that the IRE1α-Fgf regulatory circuit extends beyond AT2 cells and is present in both lung airway basal cells and pancreatic β islet cells. Finding this mechanism in multiple endodermal lineages suggests that the mechanism predates the evolution of those tissues in animals. We speculate that the IRE1α’s effects on plasticity evolved as part of its ancestral duties in preserving protein folding homeostasis. Dedifferentiation of secretory cells would have helped relieve their secretory commitments until protein folding conditions improved. Over evolutionary time, IRE1α and related pathways in the UPR/ISR became extensively cross wired into other ancient cellular stress programs (72–74), many of which are characteristically activated in DATPs (1, 2). This may explain how IRE1α and the UPR/ISR came to regulate AT2 plasticity in fibrosis, both when the primary insult is misfolded protein and in other injuries associated with fibrosis (75).

Regardless of its evolutionary origins, we and others have implicated IRE1α in the regulation of AT2 plasticity and pathological matrix deposition in pulmonary fibrosis using small molecule inhibitors and genetic approaches that block both *Xbp1* splicing and RIDD (6, 8, 46). These studies led to the suggestion that antagonism of *Xbp1* splicing would be therapeutically beneficial in pulmonary fibrosis (8). However, *Xbp1* has long been implicated in homeostasis throughout eukaryotes. In mammals, *Xbp1* is required for functions ranging from antibody production to insulin secretion, and mice lacking *Xbp1* die from failed liver development (76–78). Therapeutic approaches geared towards *Xbp1* splicing may therefore have pleiotropic effects in patients with fibrosis.

Our study addresses these potential concerns by demonstrating that inhibiting RIDD alone, while preserving *Xbp1* splicing, inhibits AT2-to-DATP differentiation and pulmonary fibrosis. Our findings do not preclude a pathological role for the active Xbp1 transcription factor in fibrosis or in other lung diseases. Nonetheless, our in vivo studies indicate that a therapeutic antifibrotic effect can be achieved in a “goldilocks zone” without interfering with *Xbp1* splicing or its attendant homeostatic functions. Compounds like PAIR2 could represent a form of precision medicine that targets a particular conformation of IRE1α critical to the fibrotic niche, while preserving IRE1α’s homeostatic functions in normal tissues.

## Methods

### Sex as a biological variable.

Both male and female mice were examined and findings were similar unless otherwise noted.

### Mouse studies.

All mice were C57BL/6 mice or congenic to that background. Littermate controls were used where possible. *Shh^Cre^* *Ern1^flox/flox^* (*IRE1α*^EpiKO^) mice and treatment with IRE1α kinase inhibitors were performed as described (6). *Xbp1*^flox/flox^ mice were a gift of A.H. Lee (79). *Sftpc*^CreERT2^ mice were a gift of H.A. Chapman (48). For bleomycin-induced fibrosis, 10–20 week-old mice were anesthetized with isoflurane and exposed to bleomycin 3 units/kg intranasally given once. For AT2 knockout studies using *Sftpc*^CreERT2^, recombination was induced by injections of tamoxifen (Sigma) in corn oil at 100 mg/kg i.p., or the equivalent volume of corn oil, for 6 total doses over the 2 weeks leading up to bleomycin exposure. For lineage tracing studies using *Sftpc*^CreERT2^, mice were injected with tamoxifen at 100 mg/kg i.p. for 2 consecutive days, followed by a 7-day chase period before exposure to bleomycin. For in vivo PAIR treatment, compound was dissolved in prewarmed 50% w/v captisol (Cydex) in water and incubated at 50^o^ C, then diluted in water to a final concentration of 3.6 mg/ml PAIR2 and 20% w/v captisol. Mice were injected with PAIR2 30 mg/kg i.p. twice daily (BID), or the equivalent volume of 20% captisol, starting on the day prior to bleomycin exposure and continuing through harvest.

### Cell isolation and culture.

For IRE1α overexpression, the cDNA encoding mouse IRE1α was integrated into TRex 293T cells (Invitrogen) as described (11) and overexpression induced with doxycycline at 1 μg/ml for 24 hours.

For organoid cultures with primary cells, mouse lungs were dissociated as described (80) and sorted for live CD45^-^ CD31^-^ EPCAM^+^ MHCII^+^ AT2 cells and CD45^-^ CD31^-^ EPCAM^-^ “triple negative” mesenchyme. Stains were used at 0.5 μl per 1 million cells: DRAQ7 (BioLegend 424001), anti-CD45 (APC-Cy7, 30-F11, BioLegend 103116, RRID:AB_1134107), anti-CD31 (APC-Cy7, 390, BioLegend 102440), anti-EPCAM (FITC, G8.8, BioLegend 118208), and anti-MHCII (PE, M5/114, Invitrogen 12532183).

The concentration of IRE1α modulators G1749, KIRA8, and PAIR2 were 2 μM unless otherwise noted. Thapsigargin (Sigma) was used at 500 nM, pemigatinib (Fisher) at 100 nM, and *m-*3M3FBS (Sigma) at 20 μM.

AT2/mesenchyme cocultures were performed as described (4). Serum-free, feeder-free AT2 cultures were performed as described [“SFFF” media (44)] except that IL-1 was omitted. For UPR induction in serum-free, feeder-free organoids, mouse primary AT2 cells were seeded in Matrigel and grown in SFFF media for 10–14 days, then pretreated with IRE1α modulators (at the concentrations listed above) for 24 hours and then stimulated with thapsigargin or G1749 (at the concentrations listed above) for 24 hours. For differentiation experiments, cells were stimulated with G1749 and/or pemigatinib (at the concentrations listed above) for 24 hours, followed by 3 days of culture in SFFF to allow time for differentiation. In differentiation experiments with PAIR2 or *m-*3M3FBS treatment, cells were continuously treated starting from the day before stimulation through the differentiation period. For Fgf10 starvation experiments, mouse primary AT2 cells were initially seeded in Matrigel with standard SFFF media with Y27632, then switched to SFFF media with the indicated concentrations of Fgf10 at the time of the first media change and subsequently cultured for an additional 12 days.

For serum-free, feeder-free airway basal cell cultures, tracheal epithelial cells were isolated as described (81) and seeded into Matrigel droplets at 5,000 cells/droplet and cultured for 12 days in DMEM/F12 Advanced (Invitrogen) supplemented with 10 μM SB431542 (Selleckchem), 10 μM Y27632 (Abcam), 1X Glutamax (Invitrogen), 1X B27 supplement (Gibco), and 10 ng/ml mouse FGF10 (R&D). Cultures were pretreated with IRE1α modulators for 24 hours followed by thapsigargin stimulation for 24 hours at the concentrations listed above. For pancreatic islet culture, freshly isolated human islets in the purity range of 80%–95% were obtained from Prodo Labs and cultured in PIM(S) medium supplemented with PIM(ABS) and PIM(G) (Prodo Labs). After overnight incubation with PIM(S) medium, G1749 (5 μM) was added to the medium for 24 hours.

### Histology.

For morphometry studies, lungs were inflated with ice-cold 4% paraformaldehyde (PFA) under constant pressure of 25 cm H2O for 5 minutes. Lungs were dissected and fixed in 4% PFA and dehydrated in 70% ethanol followed by paraffin embedding and 5 μm sectioning. For quantification of mean linear intercept (MLI), for each mouse 12 fields were quantified using ImageJ software as described (82).

Antibodies used were: rat anti-Krt8 (DSHB TROMA-I, 1:10, RRID:AB_531826), rabbit anti-Krt8 (Abcam EPR1628Y, 1:500), rat anti-Ager (R&D 4418-APC, 1:200), rabbit anti-proSPC (Millipore AB3786, 1:100, RRID:AB_91588), and chicken anti-Krt5 (BioLegend 905901, 1:100, RRID:AB_2565054). For picrosirius red staining, mouse lungs were inflated in 1.5% w/v paraformaldehyde in OCT, floated in sucrose overnight, frozen in cryomolds, and sections stained with picrosirius red (Abcam).

For cytospin quantification, organoids were released from Matrigel with 2 U/ml dispase followed by dissociation in 0.25% trypsin. Cells were resuspended in 10% bovine serum albumin and cytospun (Thermo Shandon) onto Superfrost Plus slides (Thermo). At least 180 cells were scored for each replicate.

For immunofluorescence staining, frozen sections were prepared by inflating lungs with OCT and 1.5% paraformaldehyde, followed by incubation in 4% paraformaldehyde for 1 hour, PBS for one hour, serial flotation in sucrose, and OCT embedding. Antigen retrieval was performed in Tris buffer (10 mM Tris, 1 mM EDTA, 0.05% Tween, pH 9.0) at 70 degrees for 15 minutes, followed by autofluorescence quenching in 0.1 mg/ml sodium borohydride in PBS on ice for 10 minutes. Quantification was performed by examining injured regions across 3–4 lung lobes and evaluating *Sftpc* lineage-traced cells for Krt8 staining. A total of 35 images and 3,400 cells were quantified across 8 mice. The fraction of double-positive cells (trace^+^Krt8^+^ / trace^+^) was calculated separately for each mouse.

### Gene expression and Xbp1 splicing.

For quantitative real-time PCR, Total RNA was isolated with Tri-Reagent (Ambion) and reverse transcribed with Quantitect RT kit (Qiagen). Targets were amplified with PowerUp Sybr Green (Thermo). For quantification of *Xbp1* splicing fraction, RT-PCR products were separated on 4% agarose and splice isoforms quantified as described (6). For Western blots from cultured cells, lysates were prepared in RIPA buffer (Thermo) with protease and phosphatase inhibitors (CST). For Xbp1 Western blots from *Xbp1* AT2 knockout mice, lungs were harvested 9 days after the final tamoxifen injection and epithelial cells sorted. Cells were pooled from 2 male and 4 female mice and nuclear lysates obtained using the NE-PER kit (Thermo). Blots probed for phosphorylated IRE1α (Novus NB100-2323, 1:1000), total IRE1α (Santa Cruz sc-390960, 1:1000), Gapdh (Santa Cruz sc-47724, 1:1000), Xbp1 (Biolegend 9D11A43, 1:1000), and Hdac1 (CST 5356, 1:1000). Blots were scanned using an Odyssey imager (Li-Cor).

### Single-cell and bulk sequencing analysis.

Mouse lungs were dissociated as described (80), including instillation of 1% low-melting point agarose to prevent release of cells from the conducting airways, and sorted for live CD45^-^ CD31^-^ EPCAM^+^ epithelial cells. Library preparation and sequencing was performed by the UCSF Geomics CoLabs. Single cell gel/bead emulsions were generated in on a 10X Chromium instrument (10X Genomics) and libraries constructed using the Chromium Single-Cell 3’ kit (10X Genomics), followed by sequencing on a NovaSeq 6000 (Illumina). Demultiplexing, alignment, barcode counting, and UMI counting were performed using the CellRanger pipeline on the 10x Cloud Analysis interface (10X Genomics). Normalization, scaling, dimensional reduction, and clustering were performed using Seurat4 (83). Batches and datasets were integrated using fastMNN (84). Publicly available datasets were projected using the MapQuery function in Seurat, or independently reanalyzed and reclustered followed by comparison to the bleomycin dataset using matchScore2 (85). Gene set analysis was performed using the AUCell module of SCENIC (32) using gene sets described previously (6). Differential gene expression for RIDD candidate genes was performed using the FindMarkers function in Seurat with log-fold-change threshold 0.1 and the likelihood-ratio test for single cell gene expression (test.use=’bimod’). Analysis of public bulk transcriptomic data was performed using DESeq2 (86) using the model ~Exposure.

### IRE1α* cleavage assays.

The full length *Fgfr2* CDS template was reverse-transcribed as above from AT2 cells cultured in serum-free, feeder-free organoids and amplified by Phusion high-fidelity polymerase (NEB) using primers specific to the *Fgfr2* CDS in AT2 cells. The cDNA template was transcribed using the MEGAscript T7 transcription kit (Ambion). Cleavage assays were performed with the purified IRE1α cytosolic domains (IRE1α*, Thermo) at 37° in cleavage buffer (50 mM HEPES pH 7.5, 50 mM potassium acetate, 0.05% v/v Triton X100, 10 mM magnesium chloride, and 10 mM 2-mercaptoethanol). The *Fgfr2* substrate (250 nM) was incubated with 60 nM enzyme for 60 minutes followed by precipitation in 300 mM sodium chloride and 70% ethanol, denaturation in formamide, and electrophoresis on a 2% agarose gel in tris-acetate-EDTA buffer. Products were detected by ethidium bromide staining. Kinase and RNase modulator compounds were added at the following concentrations: 500 nM KIRA8, 500 nM PAIR2, and 50 μM STF083010. Dephosphorylation of IRE1α* was performed with lambda phosphatase (NEB) according to the manufacturer’s instructions.

### Data availability.

Single cell sequencing data from this study were deposited in the Gene Expression Omnibus series GSE243124 (https://www.ncbi.nlm.nih.gov/geo/query/acc.cgi?acc=GSE243124) and series GSE243129 (https://www.ncbi.nlm.nih.gov/geo/query/acc.cgi?acc=GSE243129). Previously-published datasets used in the analysis are available from URLs or GEO accession numbers noted in the Figure legends. Values for all data points in graphs are reported in the Supporting Data Values file.

### Statistics.

Statistical methods used in each panel are specified in the figure legends. The experimental unit (e.g., individual mice or culture wells) is indicated in the respective Figure legends. Unless otherwise noted, *P* = 0.05 was used as the threshold of significance. Where multiple hypothesis corrections were performed, both significance thresholds and adjusted p values calculated by Sidak’s method are reported. Where Student’s *t* test was used, tests were 1-sided unless otherwise noted.

### Study approval.

The animal model work described in this study was approved by the Institutional Animal Care and Use Committee of the University of California, San Francisco.

## Author contributions

VCA, DS, and FRP designed the studies and wrote the manuscript. VCA, TLS, AO, LS, MEM, ISK, MSD, MT, LG, and BJB conducted experiments, acquired data, and analyzed data. DJM and BJB provided reagents.

## Supplementary Material

Supplemental data

Unedited blot and gel images

Supporting data values

## Version 1 10/15/2025

Electronic publication
