# Supplementary material for: Pharmacologic inhibition of IRE1α-dependent decay protects alveolar epithelial identity and prevents pulmonary fibrosis in mice
Source: J Clin Invest. 2025 Oct 15;135(20):e184522. doi: 10.1172/JCI184522 (PMC12520674; doi:10.1172/JCI184522)
Supplement: Supplemental data [file jci-135-184522-s267.pdf]

Pharmacologic inhibition of IRE1α-dependent decay protects alveolar epithelial identity and prevents pulmonary fibrosis in mice

Vincent C. Auyeung[1,2], Tavienne L. Steinberg[1,2], Alina Olivier[3,4], Luka Suzuki[2,3,4], Mary E. Moreno[2,3,4], Imran Khan[5,6], Michael S. Downey[1,2], Maike Thamsen[3,4], Lu Guo[1,2], Dustin J. Maly[7], Bradley J. Backes[2], Dean Sheppard[1,2,5,*], and Feroz R. Papa[1,2,3,4,*]

[1]Division of Pulmonary, Critical Care, Allergy, and Sleep Medicine, UCSF

[2]Department of Medicine, UCSF

[3]Diabetes Center, UCSF

[4]Quantitative Biosciences Institute (QBI), UCSF

[5]Cardiovascular Research Institute, UCSF

[6]Division of Neonatology, Department of Pediatrics, UCSF

[7]Department of Chemistry, University of Washington

* Corresponding authors

Supplemental figures S1-S8

Figure S1: Single cell sequencing of AT2 differentiation after fibrotic injury.

Figure S2: UPR signaling in epithelial cells from human idiopathic pulmonary fibrosis (IPF).

Figure S3: Features of fibrotic DATPs during lung development in embryonic and juvenile mice.

Figure S4: Features of fibrotic DATPs across multiple mouse models of injury.

Figure S5: Effects of IRE1α kinase modulation on AT2-to-DATP differentiation.

Figure S6: Dissection of the respective roles of *Xbp1* and RIDD in lung fibrosis in mice.

Figure S7: Targets of regulated IRE1α-dependent decay (RIDD) in AT2 cells.

Figure S8: The IRE1α-*Fgfr* regulatory circuit is conserved in additional epithelial lineages.

Figure S1

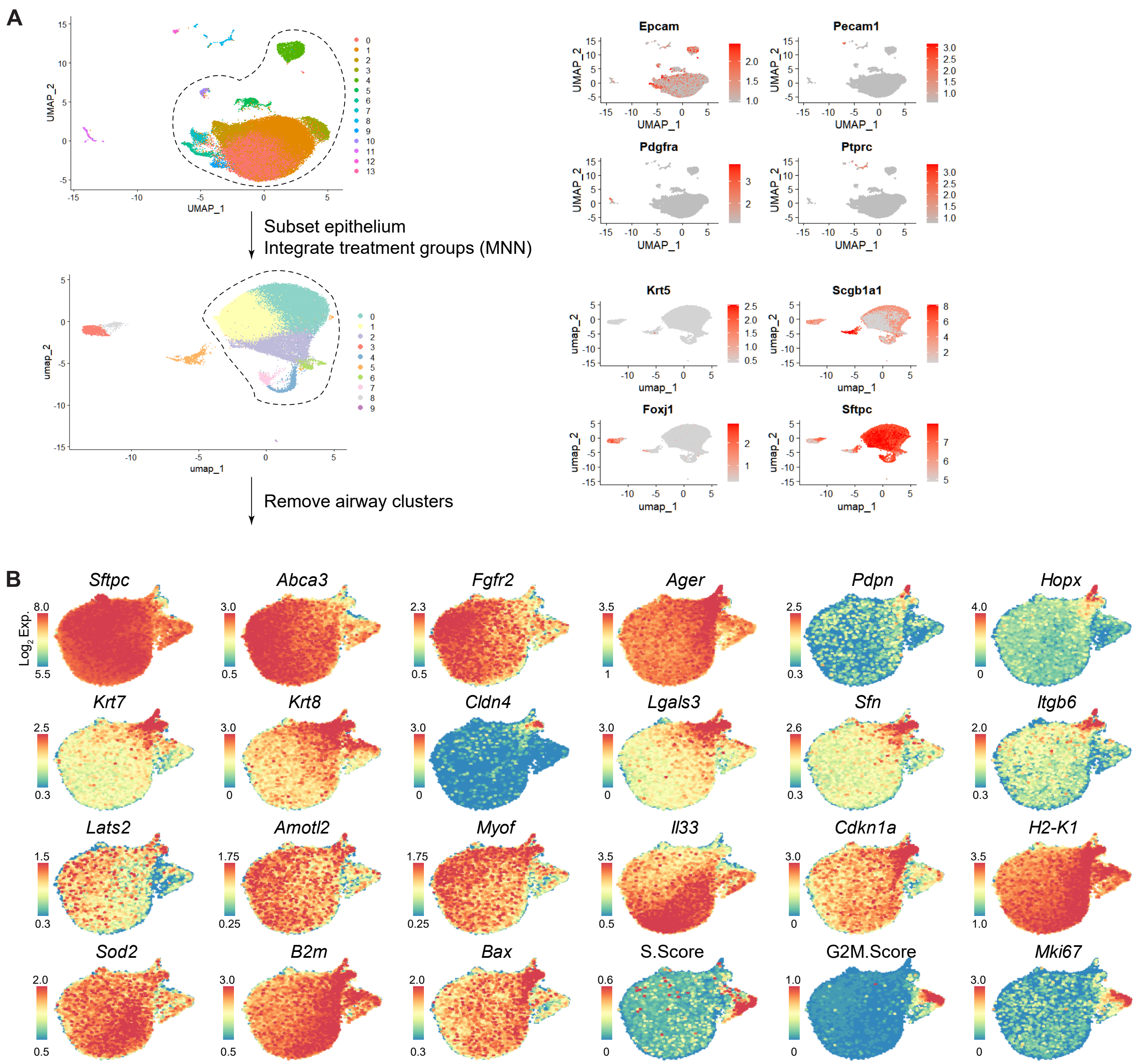

Fig S1: Single cell sequencing of AT2 differentiation after fibrotic injury. (A) Initial subsetting and integration of single cell sequencing of sorted lung epithelial cells on day 10 after saline or bleomycin exposure. (B) UMAP of subsetted cells on day 10 after saline or bleomycin exposure, with overlay of expression levels of genes marking AT2 cells, immature AT1 cells, DATPs, and genes associated with Hippo, inflammatory, and proliferative signaling.

Figure S2

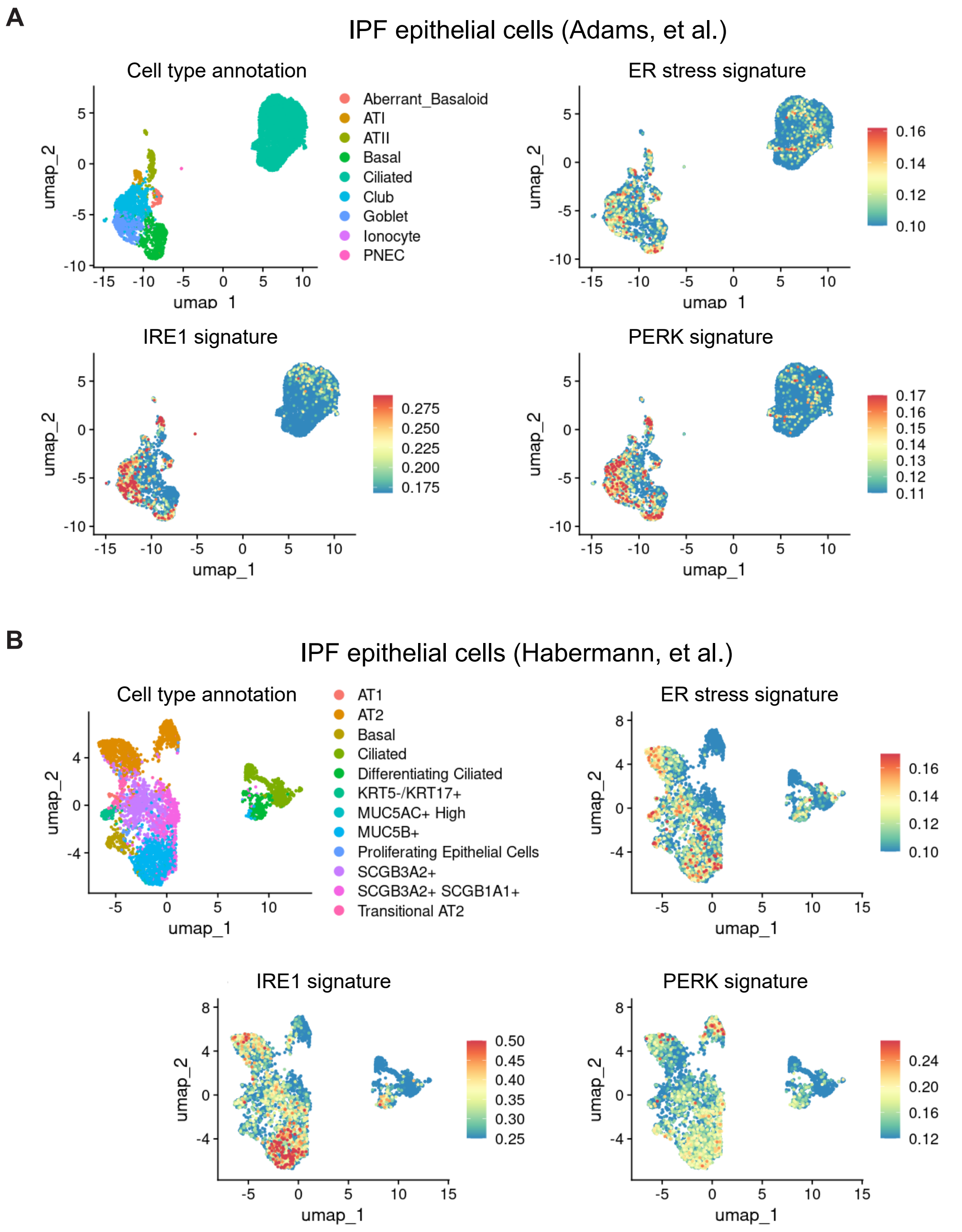

Fig S2: UPR signaling in epithelial cells from human idiopathic pulmonary fibrosis (IPF). UMAP of epithelial cells from two independent libraries from human IPF patients, GSE136831 (A) and GSE135893 (B). For each dataset, UMAP plots are overlaid with cell type annotations according to the originating publications, and inference of ER stress, IRE1α, and PERK/ISR activity based on area-under-curve (AUC) analysis of their respective signature genes. For gene set activities, colors correspond to AUC scores. Gene sets are considered inactive when AUC scores are less than the lowest score in the color bar (blue).

Figure S3

A

Normal transition

Upstream Regulator	Bias-adjusted Z-score	p-value
WNT3A	3.748	4.1E-16
Tgf beta	3.13	1.3E-10
Notch	2.355	2.1E-05
P38 MAPK	2.256	1.6E-08
FGF10	1.21	6.8E-10
TEAD2	1.688	0.00854
TEAD3	1.714	0.00926
TEAD4	1.516	4.2E-05
PTEN	2.814	3E-23
RICTOR	7.934	6.3E-45
TSC1	1.513	7.1E-08
HIF1A	2.107	6.8E-20
KCNJ2	3.634	2.5E-10

Fibrotic transition

Upstream Regulator	Bias-adjusted Z-score	p-value
DDX5	4.95	3.1E-23
RB1	3.645	1.2E-17
CDKN2A	2.356	1.3E-08
ATM	1.434	2.4E-06
CDKN1A	1.08	4.7E-10
IL33	3.932	4.1E-16
TLR4	1.66	2.7E-14
STING1	1.525	0.00027
IL1	1.024	3.5E-06
TEAD1	5.569	1.1E-36
ACLY	1.752	6.7E-05
SREBF1	3.601	4.4E-08
GDF15	1.19	0.00124
HSPA5	1.189	0.00216

B

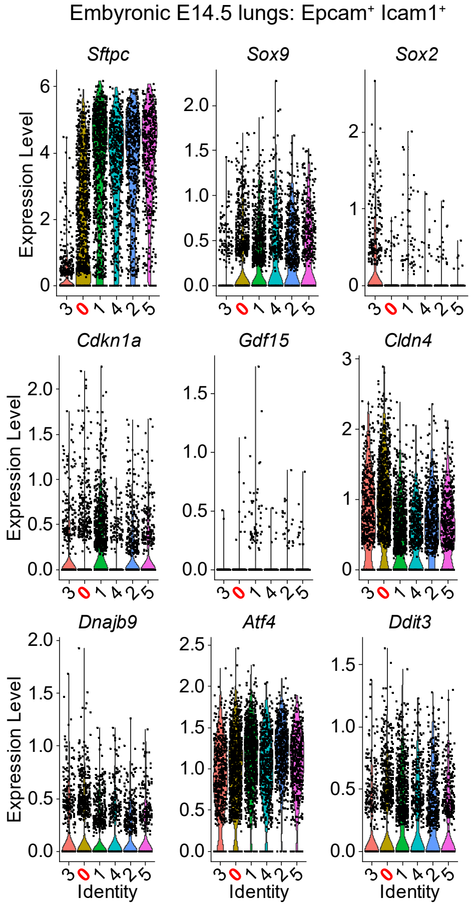

C

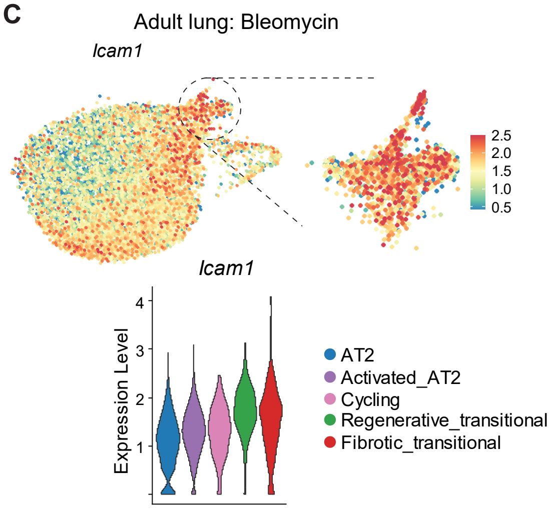

D

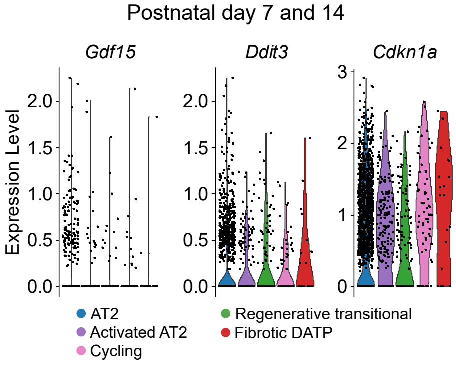

E

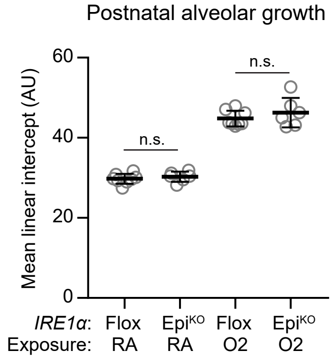

Fig S3: Features of fibrotic DATPs during lung development in embryonic and juvenile mice. (A) Violin plot of fibrotic DATP markers in cells from postnatal day 7 and day 14. (B) Violin plot of distal and proximal epithelial markers, and markers of fibrotic DATPs in sequencing of sorted Epcam+ Icam1+ cells from E14.5 embryonic lungs (GSE254356). The *Icam1*^{high} *Sftpc*^{low} *Sox2*^{low} population corresponds to cluster 0 (red). (C) *Icam1* expression in subsetted cells from adult lungs on day 10 after saline or bleomycin exposure, and detail of transitional populations. (D) Selected upstream regulators identified by Ingenuity Pathway Analysis on cells in the normal transition and the fibrotic transition. (E) Mean linear intercept morphometry of neonatal wildtype mice or mice with conditional epitheli-al IRE1α knockout (Epi^{KO}) exposed to 75% oxygen or room air. Individual mouse replicates shown with mean ±SEM. n.s. not signifcant by one-sided Student's t-test.

Figure S4

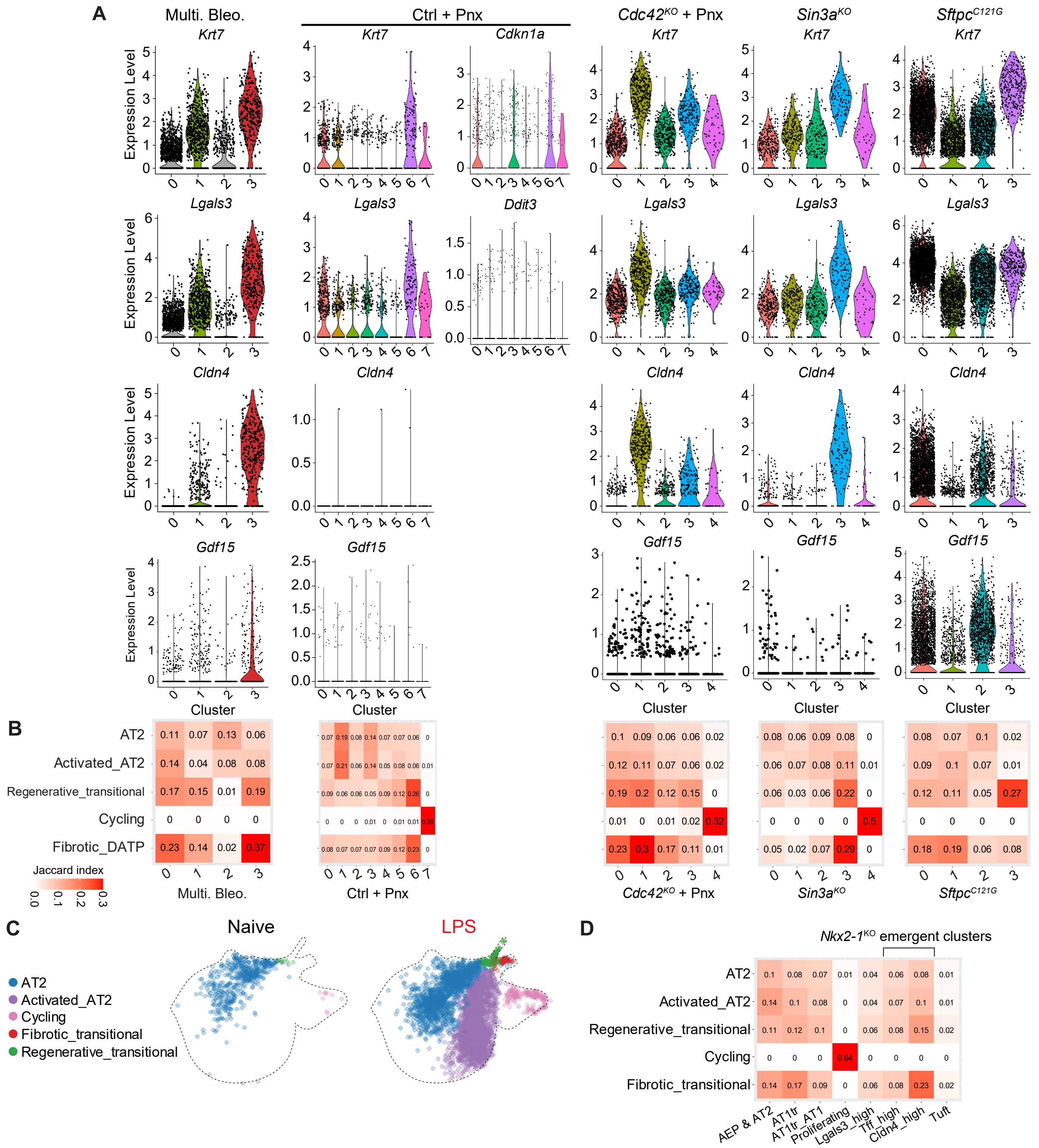

Fig S4: Features of fibrotic DATPs across multiple mouse models of injury. (A) DATP marker expression in reanalyzed and reclustered single cell sequencing from multiple-dose bleomycin (GSE243252), pneumonectomy (Pnx) in control or *Cdc42* knockout (*Cdc42*^{KO}) mice (GSE138585), conditional knockout of *Sin3a* in AT2 cells (*Sin3a*^{KO}, GSE132910), and knock-in of a mutation identified in human fibrosis patients at the Spc locus (*Sftpc*^{C121G}, GSE189479). (B) Similarity matrices comparing cell clusters in (A) to clusters identified in bleomycin injury (Fig 2A) computed using the matchScore2 package. Cluster numbers are same as in (A). (C) Integrated reference mapping of *Sftpc* lineage-traced cells from mice injured with lipopolysaccharide (LPS) (GSE113049). (D) Similarity matrix comparing emergent cell clusters in organoids grown from *Nkx2-1*^{KO} alveolar epithelial progenitors (GSE215824) to clusters identified in bleomycin injury.

Figure S5

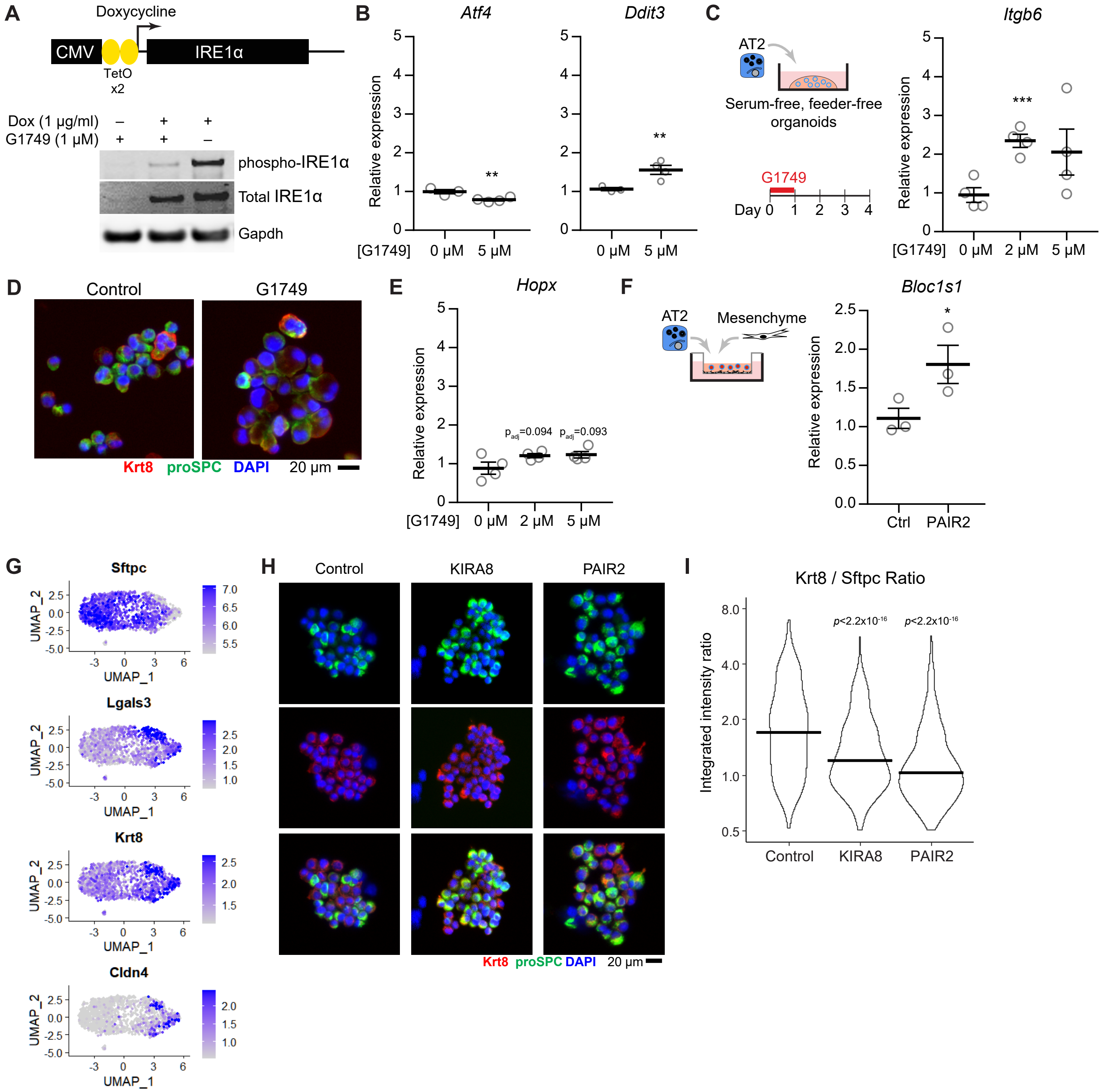

Fig S5: Effects of IRE1α kinase modulation on AT2-to-DATP differentiation. (A) IRE1α phosphorylation in 293T cells engineered to express IRE1α under doxycycline control, and treatment with the ATP-competitive activator G1749. (B) Expression of canonical PERK- and ISR-induced genes in AT2 organoids stimulated with the IRE1α activator G1749. (C) Schematic of G1749 transient exposure and expression of *Itgb6* with varying doses of G1749. (D) Representative cytospin fields for organoids pulsed with G1749 as in (C). (E) Expression of *Hopx* in organoids pulsed with G1749 as in (B). (F) Schematic of AT2 organoids co-cultured with CD45- CD31- EPCAM- mesenchyme and expression of *Bloc1s1* upon treatment with PAIR2. (G) Marker gene expression in public single-cell sequencing of organoids grown as in (F) (GSE144468). (H) Individual channel images for representative cytospin fields from organoids shown in Fig 2F. (I) Violin plots of Krt8/Sftpc intensity ratios in cytospun cells from organoids grown as in Fig 2E, based on automated segmentation and quantification of total (integrated) Krt8 and Sftpc intensity. Values plotted on a logarithmic scale with p-values calculated by Mann-Whitney test. Violin plot distributions are based on individual cells aggregated from 3 independent wells. For other panels, individual culture well replicates shown with mean ±SEM. * $p<0.05$, ** $p<0.01$, *** $p<0.001$, **** $p<0.0001$ by one-sided Student's t-test with adjusted p-values where indicated to correct for multiple hypotheses using Sidak's method.

Figure S6

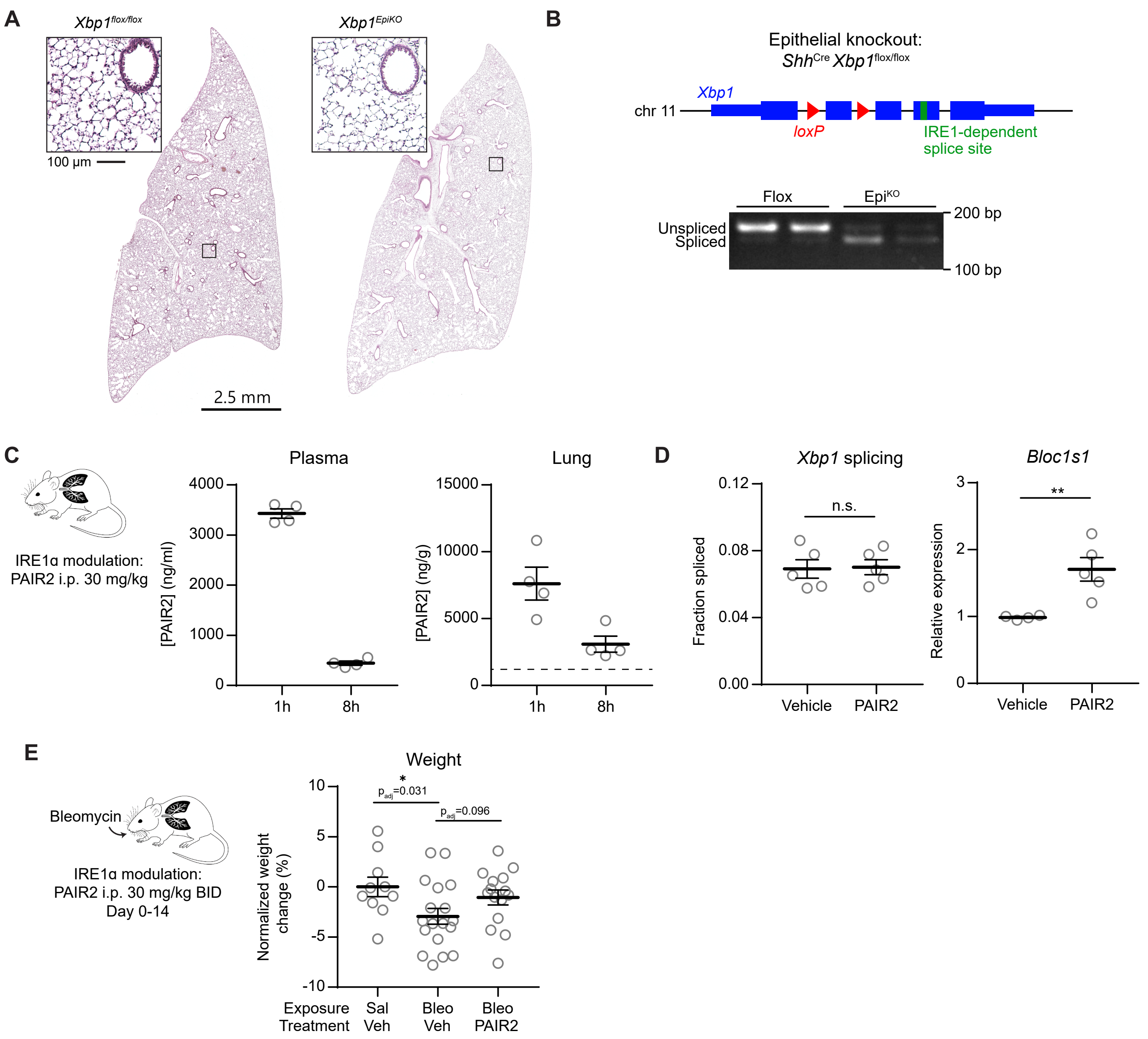

Fig S6: Dissection of the respective roles of Xbp1 and RIDD in lung fibrosis in mice. (A) Hematoxylin and eosin staining of left lungs from adult mice with *Xbp1*$^{flox/flox}$ or *Shh*^{Cre}-driven conditional knockout of *Xbp1* in the lung epithelium (*Xbp1*^{EpiKO}) (B) Diagram of the *Xbp1* locus with loxP sites flanking the second exon (red), and the non-canonical IRE1α-regulated intron in exon 4 (green), and agarose gel electrophoresis of splice isoforms of *Xbp1* detected by RT-PCR in Epcam-sorted lung epithelial cells from *Xbp1*$^{flox/flox}$ or *Xbp1*^{EpiKO} mice. (C) Plasma and lung tissue concentrations of PAIR2 at 1 and 8 hours after a single 30 mg/kg dose. The dashed line indicates the estimated effective concentration of PAIR2. (D) Lung *Xbp1* splicing fraction and *Bloc1s1* expression in mice treated with PAIR2. (E) Normalized weight change in mice exposed to bleomycin and treated with PAIR2. Individual mouse replicates shown with mean ±SEM. * $p<0.05$, ** $p<0.01$ by one-sided Student's t-test with adjusted p-values where indicated to correct for multiple hypotheses using Sidak's method.

Figure S7

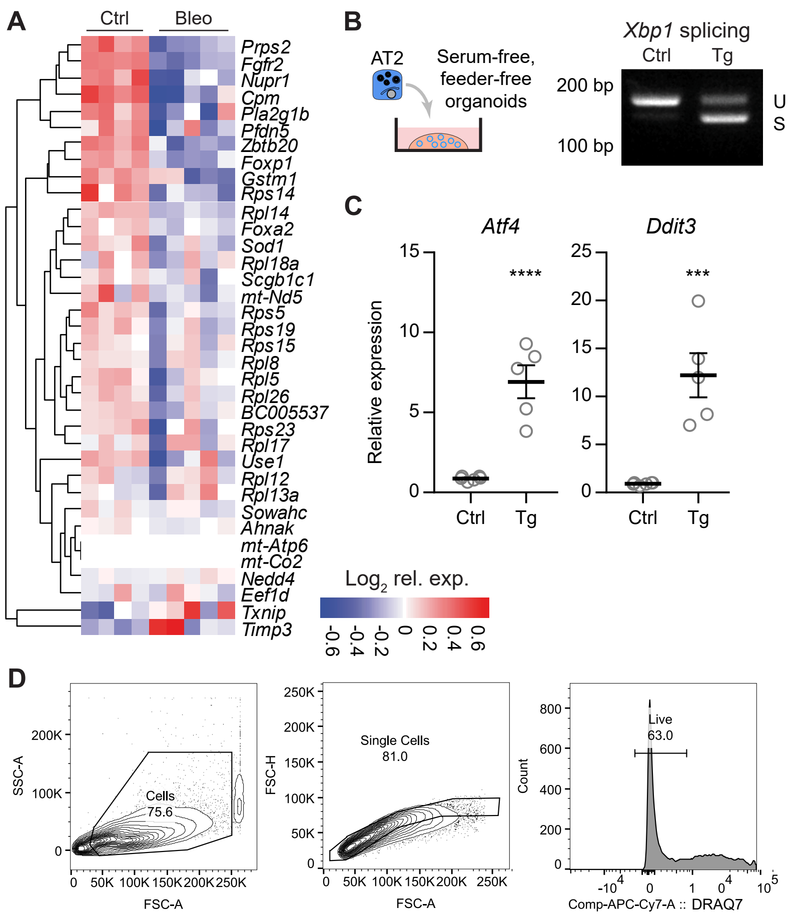

Fig S7: Targets of regulated IRE1α-dependent decay (RIDD) in AT2 cells. (A) Heatmap of candidate RIDD gene expression in bulk RNA sequencing of flow-sorted AT2 cells from mice exposed to bleomycin (GSE145771). (B) Agarose gel electrophoresis of splice isoforms of Xbp1 detected by RT-PCR in serum-free, feeder-free organoids stimulated by thapsigargin (Tg). “S” denotes the spliced and “U” the unspliced products. (C) Expression of PERK- and ISR-induced genes in AT2 organoids stimulated with thapsigargin. (D) Flow cytometry gating strategy to isolate live single cells for Fgfr2 surface expression in Fig 5F. Individual culture well replicates shown with mean ±SEM. *** p<0.001, **** p<0.0001 by one-sided Student’s t-test.

Figure S8

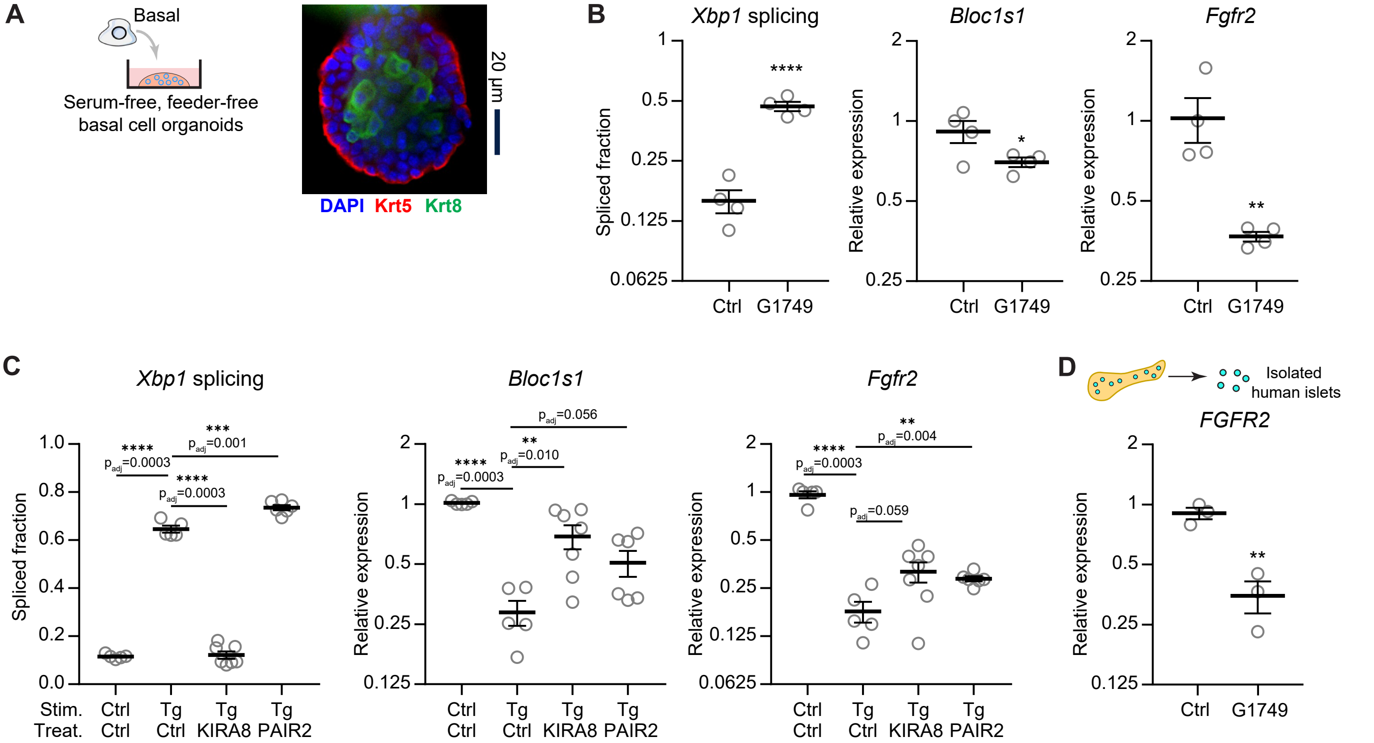

Fig S8: The IRE1α-Fgfr regulatory circuit is conserved in additional epithelial lineages. (A) Schematic of serum-free, feeder-free airway basal cell organoids. (B) Quantification of *Xbp1* splicing and expression of *Bloc1s1* and *Fgfr2* in basal cell organoids after G1749 stimulation. (C) Quantification of *Xbp1* splicing (linear scale) and RIDD target (*Bloc1s1* and *Fgfr2*) expression in serum-free, feeder-free airway basal organoids after stimulation with the ER stress agent thapsigargin (Tg) and treatment with KIRA8 or PAIR2. (D) *FGFR2* expression in primary human islets stimulated with the IRE1α-selective activator G1749. Individual culture wells replicates shown with mean ±SEM. All values plotted on a logarithmic scale unless otherwise noted. * $p<0.05$, ** $p<0.01$, *** $p<0.001$, **** $p<0.0001$ by one-sided Student's t-test with adjusted p-values where indicated to correct for multiple hypotheses using Sidak's method.
