# Supplementary material for: Pharmacologic inhibition of IRE1α-dependent decay protects alveolar epithelial identity and prevents pulmonary fibrosis in mice
Source: J Clin Invest. 2025 Oct 15;135(20):e184522. doi: 10.1172/JCI184522 (PMC12520674; doi:10.1172/JCI184522)

Full, unedited gel for Figure 4B (top left, total Xbp1)

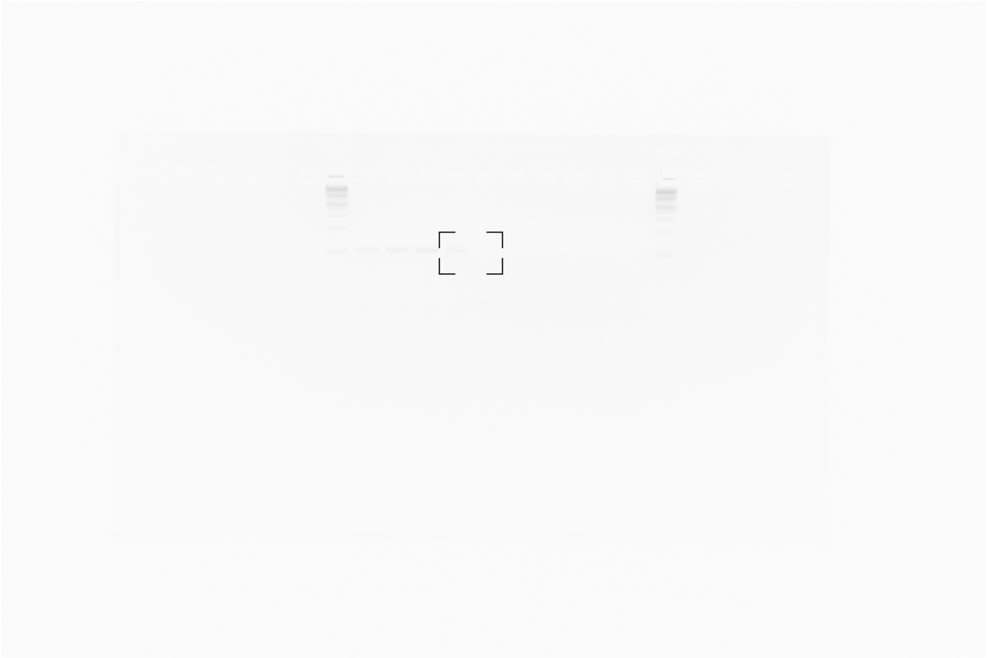

Full, unedited gel for Figure 4B (bottom left, Gapdh)

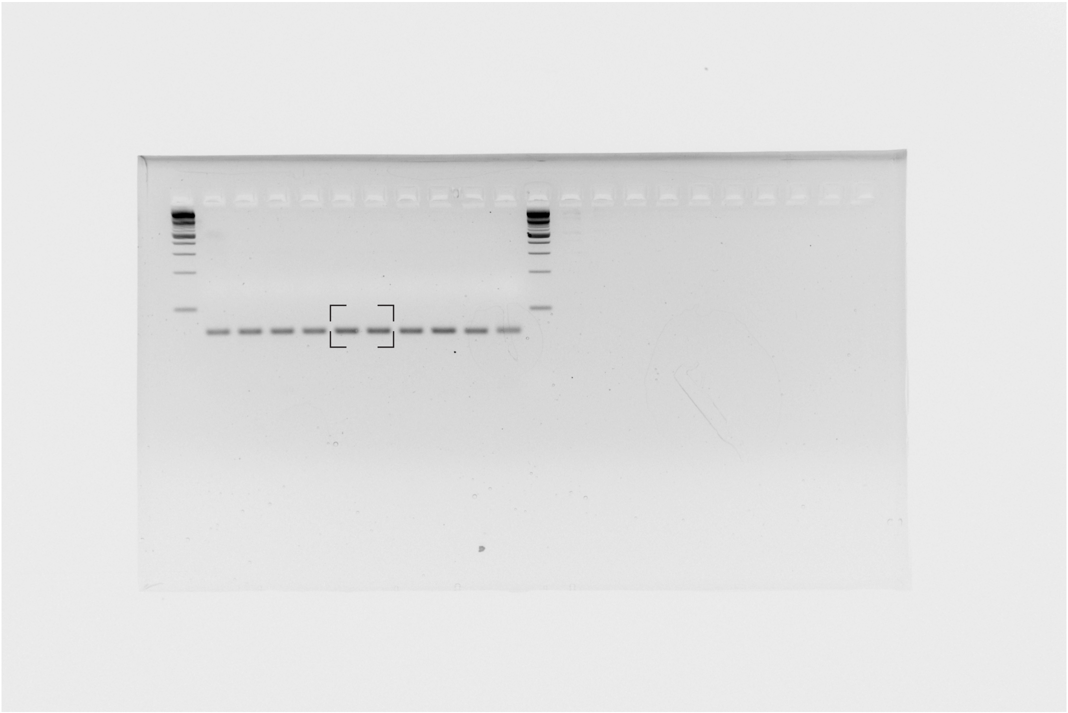

Full, unedited blot for Figure 4B (right side)

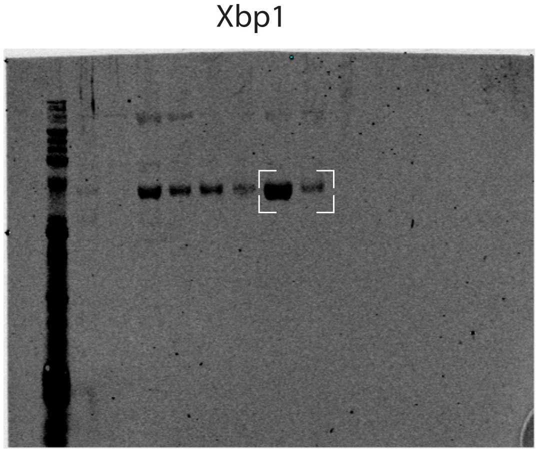

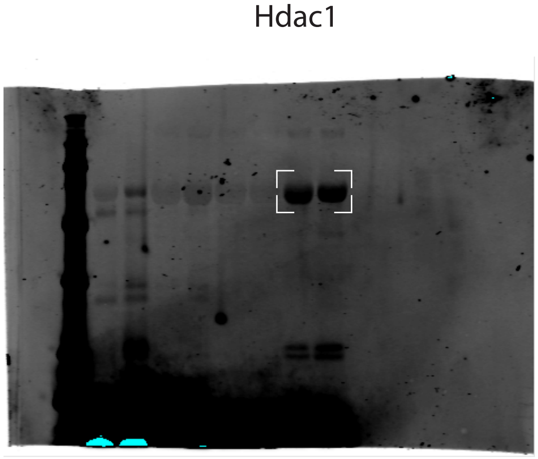

Full, unedited gel for Figure 5C

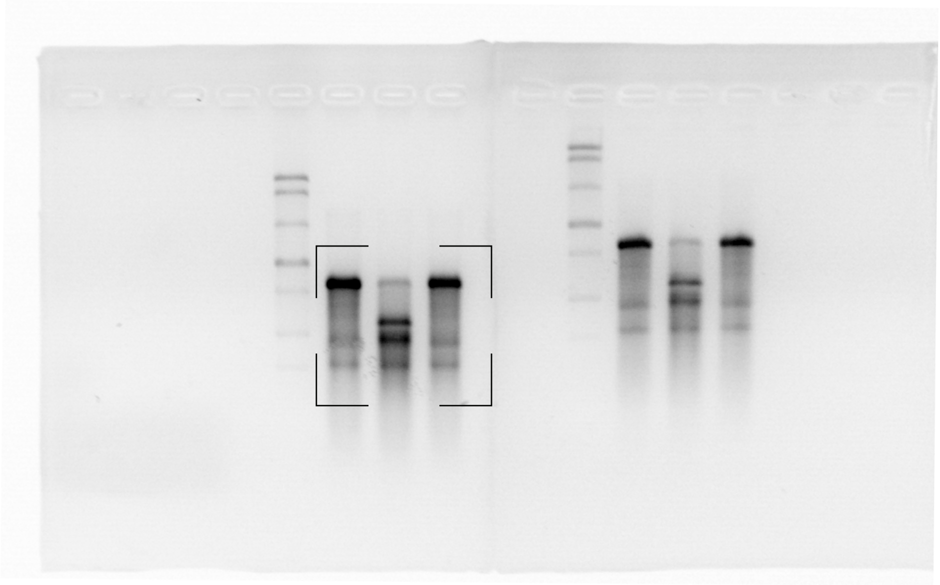

Full, unedited gel for Figure 5D

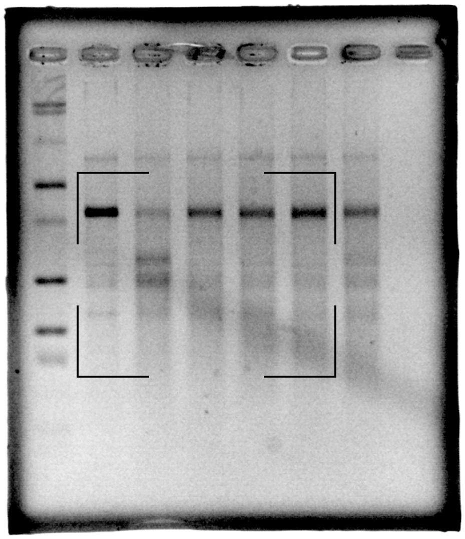

Full, unedited blots for Figure S3A

phospho-IRE1a

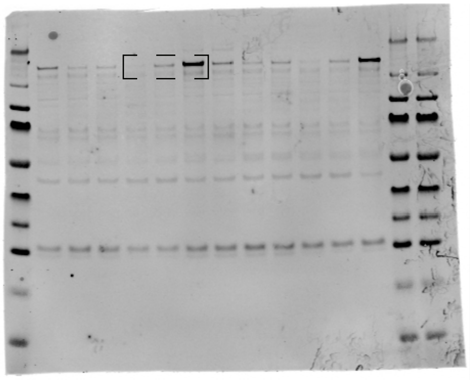

total IRE1a

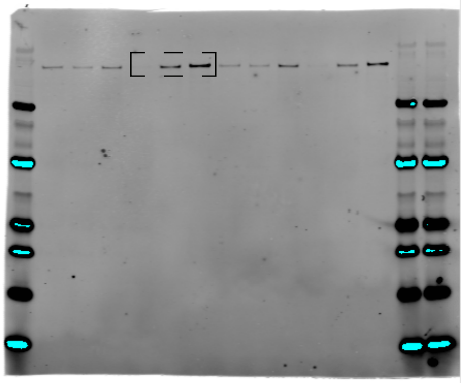

GAPDH

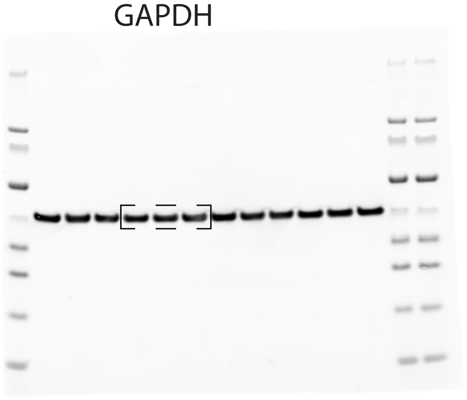

Full, unedited gel for Figure S4B

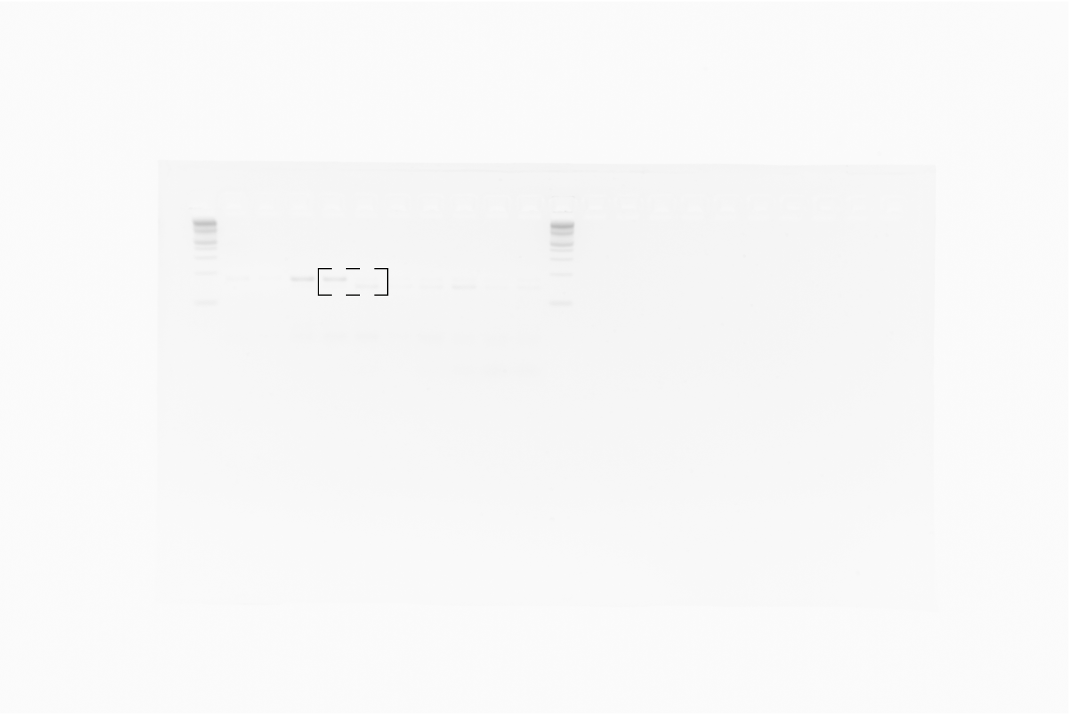

Full, unedited gel for Figure S5B

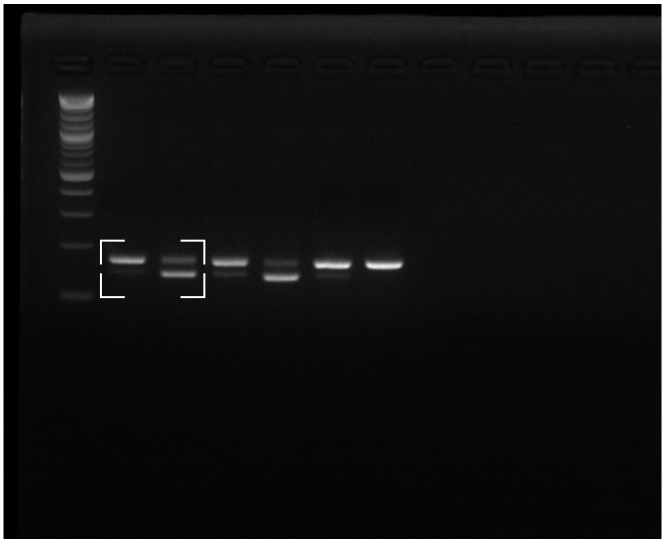

Supplement: Unedited blot and gel images [file jci-135-184522-s268.pdf]
